# Supplementary material for: Synthesis of Enantiopure Sulfoxides by Concurrent Photocatalytic Oxidation and Biocatalytic Reduction
Source: Angew Chem Weinheim Bergstr Ger. 2022 Mar 4;134(17):e202117103. doi: 10.1002/ange.202117103 (PMC10946591; doi:10.1002/ange.202117103)
Supplement: Supplementary file 1 — Supporting Information [file ANGE-134-0-s001.pdf]

Angewandte Chemie
Eine Zeitschrift der Gesellschaft Deutscher Chemiker

Supporting Information

Synthesis of Enantiopure Sulfoxides by Concurrent Photocatalytic Oxidation and Biocatalytic Reduction

S. Bierbaumer, L. Schmermund, A. List, C. K. Winkler, S. M. Glueck*, W. Kroutil*

Table of Contents

1. Materials 3
2. General procedures 3
   - 2.1. Kinetic resolution of *rac*-**1a** 3
   - 2.2. Light-dependent sulfide oxidation 3
   - 2.3. Deracemization and stereoinversion of sulfoxides 4
   - 2.4. Photocatalyst screening in the deracemization process 4
   - 2.5. Substrate screening 5
3. Preparation of the biocatalysts 5
   - 3.1. Cloning of methionine sulfoxide reductases 5
   - 3.2. Heterologous expression and cell lysis of methionine sulfoxide reductases 6
   - 3.3. Protein purification *via* nickel-affinity chromatography 7
4. Synthesis and purification of protochlorophyllide 8
5. Reference synthesis of enantiopure (*R*)-**9a** 9
6. Supplementary data 10
   - 6.1. Kinetic resolution of *rac*-**1a** 10
   - 6.2. Solvent screening of photocatalytic sulfide oxidation with **P1** 10
   - 6.3. Deracemization without photocatalyst at 385 nm 11
   - 6.4. Blank and control reactions 11
   - 6.5. Light source screening for the deracemization process with **P6** 11
   - 6.6. Protochlorophyllide concentration screening 12
   - 6.7. Redox equivalents in the cell free extract 13
   - 6.8. Time study 13
   - 6.9. Deracemization using purified paMsr 14
   - 6.10. pH screening in deracemization 14
7. Analytical methods 15
8. Calibration curves 16
9. GC-FID chromatograms 17
10. HPLC chromatograms 21
11. NMR of (*R*)-**9a** 29
12. Abbreviations 31
13. References 31

1. Materials

Chemicals were used as obtained by Sigma-Aldrich, Merck, Roth, TCI and PorphyChem without further purification except stated otherwise. Compounds *rac*-**1a**, (*R*)-**1a**, (*S*)-**1a**, **1b**, **1c**, **P1**, **P2b**, **P5**, **P7** and *rac*-**2a** were purchased from Sigma-Aldrich. Compounds **P2a**, **P3** and **P4** were obtained from PorphyChem. DTT was purchased from Roth. Compounds *rac*-**4a**, *rac*-**6a** and *rac*-**7a** were purchased from Combi Blocks and compound *rac*-**3a** was obtained from the company Enamine. Compound *rac*-**5a** originates from Apollo Scientific, and rifampicin was purchased from TCI. **P6** was isolated and purified as described in section 4.

Note for photon fluxes that the actinometry measurements were done on a reaction volume of 1 mL, whereas all experiments described herein were run on a 0.5 mL scale, except stated otherwise.[1]

2. General procedures

2.1. Kinetic resolution of *rac*-1a

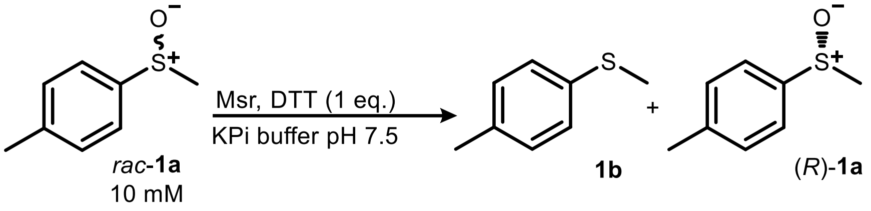

Scheme S1. Kinetic resolution of *rac*-**1a** using lyophilized cell free extracts of Msrs or purified paMsr.

For the kinetic resolution of *rac*-**1a**, lyophilized cell free extract of paMsr, pmMsr and MsrA (5 mg) respectively, was rehydrated in KPi buffer (50 mM, pH 7.5, 50 µL) for 20 min. In the meantime, KPi buffer (50 mM, pH 7.5, filled to 0.5 mL reaction volume), *rac*-**1a** (100 mM stock in KPi buffer, pH 7.5, 50 µL, 10 mM final concentration) and DTT (1 M stock in KPi buffer, pH 7.5, 5 µL, 10 mM final concentration) were mixed in a screw cap vial (1.5 mL). The reaction was started by adding the hydrolyzed CFE preparation of the enzyme (paMsr, pmMsr, MsrA, 50 µL) or purified enzyme (paMsr, 25 mg/mL stock, 20 µL). The reaction mixtures were incubated in an Eppendorf shaker (30 °C, 500 rpm, 1 h).

For work-up a spatula tip of NaCl was added to the reaction mixture prior to extraction with EtOAc (500 µL) containing decane (10 mM) as internal standard. After mixing and centrifugation (14680 rpm, 3 min), the organic phase (300 µL) was transferred to a fresh vial containing Na_2SO_4 for drying. The extraction step was repeated (500 µL EtOAc without internal standard) and the combined and dried organic phases were centrifuged (14680 rpm, 5 min) and transferred to a glass crimp vial (1.5 mL) for analysis on GC-FID.

2.2. Light-dependent sulfide oxidation

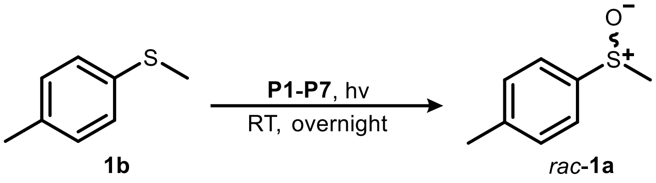

Scheme S2. Photocatalytic sulfide oxidation.

The photocatalyst was weighted into a glass vial (**P1**-**P5** and **P7**, 0.5-1 mg) or added from a MeOH stock (**P6**, 16.3 mM stock, 10 µL, 0.33 mM final concentration), dissolved in KPi buffer (pH 6.5, 50 mM, 0.5 mL) followed by the direct addition of **1b** (1.35 µL, 20 mM). The vials (1.5 mL) were then illuminated with 405 nm (**P1**-**P5** and **P7**) or 455 nm (**P6**) and intensity (0.99 µmol photons s^{-1} and 0.036 µmol photons s^{-1}, respectively) at room temperature (25 °C, 500 rpm). For quantification, the reaction mixture was transferred to a fresh microcentrifuge tube (1.5 mL) and extracted with EtOAc (2x 500 µL). The combined organic phases were dried over anhydrous Na_2SO_4 and analyzed on GC-FID.

2.3. Deracemization and stereoinversion of sulfoxides

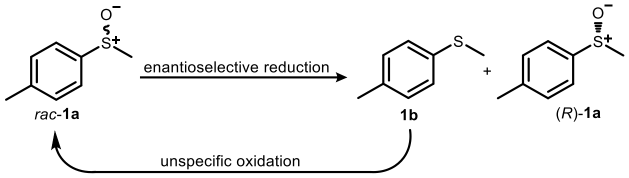

Scheme S3. Cyclic deracemization process of *rac*-sulfoxides based on an enantioselective biocatalytic reduction followed by a photocatalytic sulfide oxidation.

For the deracemization as well as stereoinversion reactions, KPi buffer (pH 6.0, 500 mM stock, 50 µL, 50 mM final concentration) was pipetted into a screw cap glass vials (1.5 mL), followed by the addition of **1a** [100 mM stock in H_2O, 50 µL, 10 mM final concentration of *rac*-**1a** or (*S*)-**1a**, 100 mM stock in H_2O, 25 µL, 5 mM final concentration], DTT (50 mM final concentration, 1 M stock in H_2O) and **P6** (2.57 mM in MeOH, 10 µL, 51 µM final concentration). The reaction was started by the addition of CFE (5 mg of paMsr/pmMsr/MsrA dissolved in H_2O, respectively). The reaction mixture was filled to 0.5 mL using H_2O.

Work-up for GC analysis was done as described in section 2.1.

2.4. Photocatalyst screening in the deracemization process

The photocatalyst was weighted into the glass vial (**P2b**, **P3**, **P5**, **P7**, 0.5-1 mg) or added from a MeOH stock (**P6**, 16.3 mM stock, 10 µL, 0.33 mM final concentration), **1a** [100 mM stock in KPi buffer (pH 7.5, 50 mM), 50 µL, 10 mM final concentration] and DTT [1 M stock in KPi buffer (ph 7.5, 50 mM), 10 µL, 20 mM final concentration] were added. The reaction mixture was filled to 0.5 mL using KPi buffer (pH 7.5, 50 mM). The reaction was started by the addition of paMsr CFE [10 mg dissolved in 300 µL KPi buffer (pH 7.5, 50 mM)]. The vials were then placed in the custom photoreactor developed by C. K. Winkler *et al.*[1] and illuminated with the respective wavelength and intensity as stated in Table S1 for 24 h.

For analysis, the samples were extracted twice with EtOAc (2x 500 µL) containing decane (10 mM) as internal standard. The combined organic phases were dried with Na_2SO_4 and analyzed on GC-FID.

Table S1. Illumination conditions for the photocatalysts screening in the deracemization process.

entry	PC	wavelength [nm]	effective photon flux [µmol s^{-1}]
1	**P2b**	405	0.165
2	**P3**	405	0.165
3	**P5**	455	0.180
4	**P6**	455	0.036
5	**P7**	528	0.075

Note for photon fluxes, that the actinometry measurements were measured on 1 mL reactions, in contrast to the reaction volume of 0.5 mL applied herein.[1]

2.5. Substrate screening

For the deracemization of substrates **2a**-**9a**, KPi buffer (pH 6.0, 0.5 M stock, 50 µL, 50 mM final concentration) was pipetted into a screw neck glass vial followed by the addition of substrates **2**-**8** (100 mM stock in H_2O, 50 µL, 10 mM final concentration), DTT (1M stock in H_2O, 25 µL, 50 mM) and **P6** (2.57 mM stock in MeOH, 10 µL, 51 µM final concentration). Substrate **9a** was from a MeOH stock (1000 mM stock in MeOH, 5 µL, 10 mM final concentration). The reactions were started by adding CFE of paMsr (5 mg dissolved in 50 µL H_2O). The vials were then placed in the custom photoreactor developed by C. K. Winkler *et al.*[1] at 30 °C, 400 rpm and illuminated at 455 nm (0.036 µmol photons s^{-1}) for 24 h or as indicated otherwise.

Substrate **8a** required the application of purified protein (20 µL of a 24 mg/mL stock, 480 µg) and the addition of triton X-100 (10% v/v stock, 1.5 µL, 0.03% v/v final concentration).

The conditions for substrate **10a** were slightly varied due to its poor solubility and light instability. For the deracemisation the substrate **10a** was dissolved in KPi buffer (50 mM substrate in 0.5 M KPi buffer pH 8.0, final pH 7.5). Water (up to 500 µL), substrate (50 mM in KPi buffer, 10 mM final substrate concentration, 100 mM final buffer concentration), DTT (1M stock in H_2O, 25 µL, 50 mM) and **P6** (2.57 mM stock in MeOH, 10 µL, 51 µM final concentration) were pipetted into a screw neck glass vial before the reaction was started by the addition of paMsr CFE (5 mg dissolved in 50 µL H_2O). The vials were then placed in the custom photoreactor developed by C. K. Winkler *et al.*[1] at 30 °C, 400 rpm and illuminated at 455 nm (0.018 µmol photons s^{-1}) for 5 min.

For work-up, the samples were extracted with EtOAc (500 µL) without internal standard. After vortexing the samples were centrifuged (14680 rpm, 2 min) and the organic phase (300 µL) was transferred to a fresh microcentrifuge tube containing Na_2SO_4 for drying. The extraction step was repeated, the dried combined organic phases were centrifuged (14680 rpm, 5 min) and transferred to a glass vial for HPLC analysis.

3. Preparation of the biocatalysts

3.1. Cloning of methionine sulfoxide reductases

The enzymes were selected based on a literature search. The corresponding genes were ordered from General Biosystems as optimized sequence for overexpression in *E. coli* and cloned into a pET28a vector either by restriction enzyme cloning (MsrA, restriction sites EcoRI and XhoI) or *via* TEDA[2] cloning (paMsr, pmMsr). Successful cloning was confirmed by sequencing.

paMsr:[3]

Optimized DNA sequence:

ATGGTGCTGCGCAGTCAGATTCTGGTGAATAAGGATGTGCTGCCGACCGCCGAACAGGCACTGCCGGGTCGT
GCAGAAGCAATGCCGGTGGCAGATACCCATTATGTGAATGGTAATCCGATTAAGGCACCGTTTCCGGCCGGCC
TGCAGCAGGCAGTTTTTGGCCTGGGTTGTTTTTGGGGTGCAGAACGTCGCTTTTGGCAGCAGCCGGGCGTGTT
TAGTACCGCCGTTGGCTATGCCGGTGGCCTGACCCCGAATCCGACCTATGAAGAAGTGTGTAGTGGCCTGACC
GGCCATACCGAAGTTGTGCTGGTTGTTTTTGATCCGCAGCAGACCAGCTTTGAAGCACTGCTGAAAGTTTTCTG
GGAAGTGCATAATCCGACCCAGGGCATGCGTCAGGGCAATGATCAGGGTACCCAGTATCGTAGCGCAATCTA
TTGCCAGGATGATGCCCAGCTGAGCGCCGCCAAAGCAAGCCAGGCACGTTTTCAGGCAGAACTGGATAAAGC
CGGTGTTGGTAGCATTACCACCGAAATTGCCGAAGCCCCGACCTTTTATTATGCAGAAACCTATCATCAGCAGT
ATCTGGCAAAAAATCCGGGCGGTTATTGTGGTCTGGGCGGCACCGGCGTTTGCCTGCCTCCTGAAAGCTAA

Amino acid sequence:

MVLRSQILVNKDVLPTAEQALPGRAEAMPVADTHYVNGNPIKAPFPAGLQQAVFGLGCFWGAERRFWQQPGVF
STAVGYAGGLTPNPTYEEVCSGLTGHTEVVLVVFDPQQTSFEALLKVFWEVHNPTQGMRQGNDQGTQYRSAIYC
QDDAQLSAAKASQARFQAELDKAGVGSITTEIAEAPTFYYAETYHQQYLAKNPGGYCGLGGTGVCLPPES*

pmMsr:[4]

Optimized DNA sequence:

ATGGTGCTGCGTAGTGAAATTCTGGTTAATAAGAATGTGATGCCGACCGCCGAACAGGCACTGCCGGGTCGTGAAACCCC
GATGAGTCTGCCGGAATTTCATTATGTTTTTAAAGATACCCCGCTGCTGGGTCCGTTTTTCGAAGGCGCCATTGATTTTGCC
ATTTTTGGTCTGGGTTGTTTTTGGGGTGCAGAACGCCGCTTTTGGCAGCGTGAAGGCGTTGTTAGTACCGTGGTTGGCTAT
GCAGGTGGTTTTACCCCGCATCCGACCTATGAAGAAGTTTGTAGTGGTCTGACCGGCCATACCGAAGTGGTGCTGGTTGT
GTTTGATAAAGATCGTGTGAGTTATCGTGAACTGCTGGCAATGTTTTGGGAACTGCATAATCCGACCCAGGGCATGCGTCA
GGGCAATGATATTGGCACCCAGTATCGCAGTGCCATCTATTGTACCAGTCCGGAACAGCTGGAACAGGCCAAAGCAAGCC
GTGATGCATTTCAGGCCGAACTGAGTAAAGCCGGCTTTGGTGAAATTACCACCGAAATTGATCAGGCCCCGACCGTTTATT
TTGCAGAAGCCTATCATCAGCAGTATCTGGCCAAAAATCCGGATGGCTATTGTGGTATTGGCGGTACCGGCGTTTGCCTGC
CGCCGAGTCTGCAGGGTAATTAA

Amino acid sequence:

MVLRSEILVNKNVMPTAEQALPGRETPMSLPEFHYVFKDTPLLGPFFEGAIDFAIFGLGCFWGAERRFWQREGVVS
TVVGYAGGFTPHPTYEEVCSGLTGHTEVVLVVFDKDRVSYRELLAMFWELHNPTQGMRQGNDIGTQYRSAIYCTS
PEQLEQAKASRDAFQAELSKAGFGEITTEIDQAPTVYFAEAYHQQYLAKNPDGYCGIGGTGVCLPPSLQGN*

MsrA:[5]
Uniprot: P0A744

Optimized DNA sequence:

ATGAGCCTGTTTGATAAAAAGCATCTGGTTAGCCCGGCAGATGCCCTGCCGGGTCGCAATACCCCGATGCCGG
TGGCCACCCTGCATGCAGTGAATGGTCATAGTATGACCAATGTTCCGGATGGCATGGAAATTGCCATTTTTGCA
ATGGGTTGCTTTTGGGGCGTGGAACGTCTGTTTTGGCAGCTGCCGGGTGTGTATAGTACCGCCGCCGGCTATA
CCGGTGGTTATACCCCGAATCCGACCTATCGTGAAGTGTGCAGTGGTGACACCGGTCATGCAGAAGCCGTTCG
TATTGTTTATGATCCGAGCGTTATTAGCTATGAACAGCTGCTGCAGGTGTTTTGGGAAAATCATGATCCGGCCC
AGGGTATGCGTCAGGGCAATGATCATGGCACCCAGTATCGTAGTGCCATCTATCCGCTGACCCCGGAACAGGA
TGCAGCAGCCCGCGCCAGTCTGGAACGTTTTCAGGCCGCCATGCTGGCAGCCGATGATGATCGTCATATTACC
ACCGAAATTGCAAATGCAACCCCGTTTTATTATGCCGAAGATGATCATCAGCAGTATCTGCATAAAAATCCGTA
TGGCTATTGCGGTATTGGTGGCATTGGTGTTTGTCTGCCGCCGGAAGCCTAA

Amino acid sequence:

MSLFDKKHLVSPADALPGRNTPMPVATLHAVNGHSMTNVPDGMEIAIFAMGCFWGVERLFWQLPGVYSTAAGY
TGGYTPNPTYREVCSGDTGHAEAVRIVYDPSVISYEQLLQVFWENHDPAQGMRQGNDHGTQYRSAIYPLTPEQDA
AARASLERFQAAMLAADDDRHITTEIANATPFYYAEDDHQQYLHKNPYGYCGIGGIGVCLPPEA*

3.2. Heterologous expression and cell lysis of methionine sulfoxide reductases

The recombinant plasmid was transformed into *E. coli* BL21(DE3) for heterologous expression of the corresponding enzyme. For the overnight culture, lysogeny broth (LB, 10 mL) containing kanamycin (50 mg/mL, 10 µL, 50 µg/mL) was inoculated from an agar plate or from glycerol stock and incubated overnight (37 °C, 120 rpm). For the main culture, sterile LB medium in a non-baffled flask (700 mL in a 2 L flask), containing kanamycin (50 mg/mL, 700 µL, 50 µg/mL) was inoculated with the *E. coli* overnight culture (1% v/v). The culture was incubated at 37 °C and 120 rpm until an OD_{600} of 0.4-0.6

was reached, followed by induction adding IPTG (0.2 mM for paMsr and pmMsr, 0.5 mM for MsrA). Further incubation took place at 20 °C and 120 rpm overnight. The cell culture was harvested by centrifugation (5000 rpm, 20 min, 4 °C), the obtained supernatant discarded, and the remaining pellet washed with KPi buffer (50 mM, pH 7.5, 30 mL). The suspension was centrifuged (8 °C, 4500 rpm, 20 min), the supernatant discarded and the remaining pellet either lysed or shock frozen in liquid nitrogen and stored at -20 °C until further usage.

Lysis of the resuspended whole cells in either KPi buffer (50 mM, pH 7.5, 10 mL/g cell pellet; when the CFE was required) or in lysis buffer (see Table S2; for further purification), was done on ice by ultrasonication (three times 2 min 30 sec, 30% amplitude, 2.0 sec pulse on, 4.0 sec pulse off; 1 min pause on ice between the sonications; Digital sonifier, BRANSON). The cell suspension was centrifuged (20 min, 18 000 rpm, 4 °C) and the clear slightly yellow cell free extract (CFE) was filtered (0.45 µm syringe filter) and lyophilized or stored on ice for further protein purification using metal ion affinity chromatography.

Successful expression of soluble enzyme was verified by SDS page analysis (Figure S1).

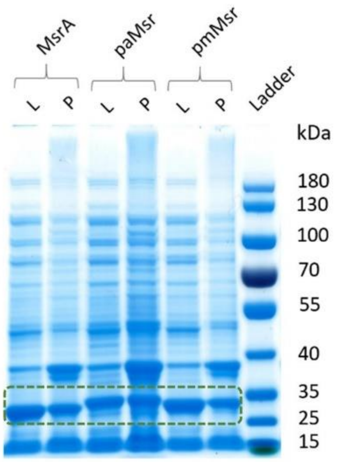

Figure S1. SDS-Page to verify expression of methionine sulfoxide reductases. L = cell free extract, P = insoluble fraction.

3.3. Protein purification *via* nickel-affinity chromatography

The His_6-tag containing paMsr was purified by nickel affinity chromatography using a HisTrap™ FF 5 mL column (GE HEALTHCARE) in combination with Äkta™ pure (GE Healthcare). The purification was performed at 4 °C using a flow rate of 5 mL/min. The column was equilibrated with 5 column volumes (CV) of lysis buffer (see Table S2) prior to loading the CFE onto the column at a flow rate of 2 mL/min. The column was washed with 5 CV lysis buffer (5 mL/min) containing 1% elution buffer (see Table S2) before the elution buffer was applied in a gradient (1-100% elution buffer) in order to elute the protein. As the eluted protein was colorless, absorption at 280 nm was used to identify the fractions (each 5 mL) containing protein. The protein containing fractions were combined and concentrated to 2.5 mL using Vivaspin® 20 mL (SARTORIUS, 10 kDa cut-off). To exchange the buffer, a Sephadex G-25 PD10 desalting column (GE Healthcare) was equilibrated with KPi buffer (50 mM, pH 7.5). The concentrated protein solution (2.5 mL) was loaded onto the column and eluted with 3.5 mL KPi buffer (50 mM, pH 7.5). The final enzyme solution was aliquoted (100 µL portions in pcr tubes), shock frozen in liquid nitrogen and stored at -20 °C.

Table S2. Buffer compositions used for protein purification.

buffer	composition
KP_i buffer	total 50 mM: 7.58 g/L Na_2HPO_4 x 2 H_2O (42.59 mM), 1.01 g/L KH_2PO_4 (7.41 mM), pH 7.5
lysis buffer	50 mM TRIS/HCl, 150 mM NaCl, 20 mM imidazole, pH 7.5
elution buffer	50 mM TRIS/HCl, 150 mM NaCl, 500 mM imidazole, pH 7.5

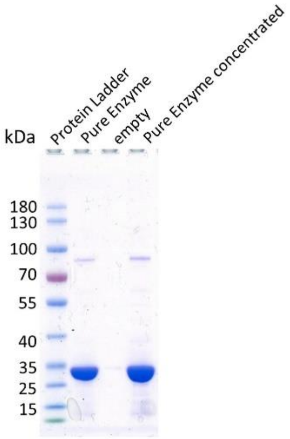

Figure S2. SDS-Page of purified paMsr.

4. Synthesis and purification of protochlorophyllide

Protochlorophyllide **P6** (pchlide) was isolated and purified from *Rhodobacter capsulatus* ZY5 strain as described in literature (cf. Figure S3, B-G).[3] Cells from a *Rhodobacter capsulatus* ZY5 glycerol stock were plated on a VN-agar plate (10 g/L yeast extract, 1 g/L K_2HPO_4, 0.5 g $MgSO_4$, pH 7.0) containing rifampicin [25 µg/mL, rifampicin stock in DMSO (50 mg/mL)]. The agar plate was incubated at 32-36 °C for 40 h, before VN-medium (100 mL, containing 25 µg/mL) was inoculated with several colonies. The preculture was incubated at 34 °C for 30 h. For the main culture, the preculture was added to 1 L of VN-medium (containing 25 µg/mL rifampicin) and additionally three white polyurethane foam bungs (height/diameter 50/35 mm) were added. Further incubation was done at 34 °C and 120 rpm for another 48 h. The dark green foam bungs were exchanged after 24 h and dried at room temperature in the dark. The absorbed dark green pchlide was washed from the 6 foam bungs with MeOH (~800 mL), which was then removed under reduced pressure. The crude pchlide was then resuspended in acetone (800 mL) supplemented with 1.5% (v/v) MeOH.

A CM Sepharose Fast Flow column (Sigma Aldrich) was prepared by washing the resin (50-75 mL) with deionized water (dH_2O, 500 mL). The resin was then resuspended in acetone, stirred, and dried under suction in a Buchner funnel, which was repeated three times. The final slurry was resuspended in acetone (100 mL) and poured into a glass column (5 cm width).

The pchlide suspension was loaded onto the column. The column was then washed with acetone (800 mL) until the flowthrough became clear. In order to remove phytol or pheophorbide the column was subsequently washed with acetone containing MeOH (5% v/v, 500 mL). The purified pchlide was eluted from the column with MeOH in acetone (25% v/v, 500 mL) and concentrated to 50 mL under reduced pressure. The pchlide solution was aliquoted (1 mL fractions in 1.5 mL microcentrifuge tubes) and the remaining solvent was removed under pressure. The aliquotes were stored at -21 °C until further usage.

A yield of 37.9 mg pchlide was determined by recording a UV-Vis spectrum (Figure S3, A) on the photometer (Cary 60 UV-Vis, Agilent Technologies) and calculation following Lambert Beer`s Law with ε_{630}= 23.95 mM^{-1} cm^{-1}.[4]

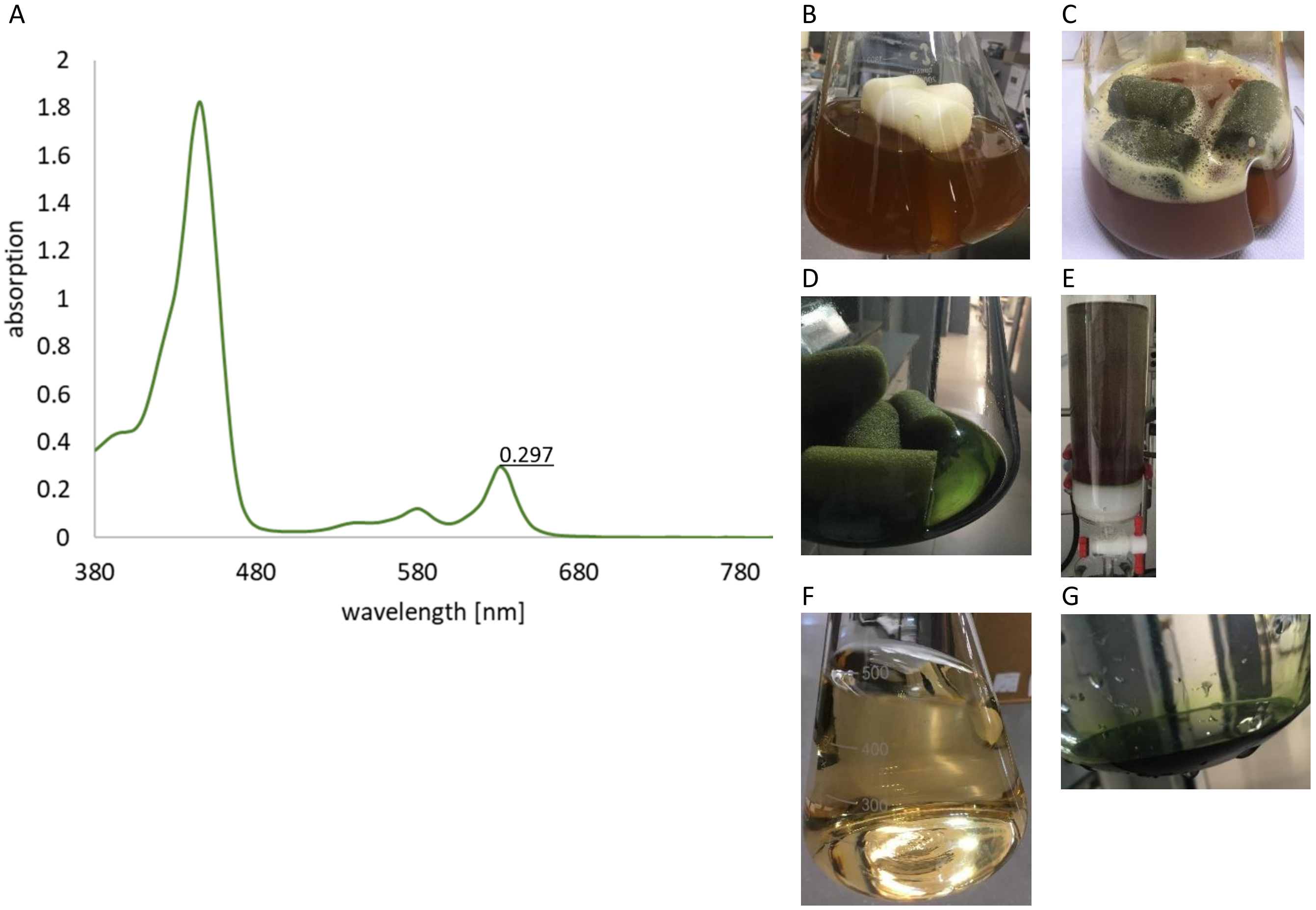

Figure S3. A: UV-Vis absorption spectrum of the purified pchlide (ε_{630}= 23.95 mM^{-1} cm^{-1})[4]; B: *Rhodobacter capsulatus* ZY5 culture with white polyurethane foam bungs; C: *Rhodobacter capsulatus* ZY5 culture incubated for 24 h with the pchlide absorbed onto the foam bungs; D: Pchlide washed from the foam bungs with MeOH; E: CM Sepharose Fast Flow column with the pchlide suspension loaded; F: Flowthrough containing carotenoids; G: Final purified concentrated pchlide solution.

5. Reference synthesis of enantiopure (*R*)-9a

In order to assign the absolute configuration of the deracemized **9a**, a reference compound was synthesized by biocatalytic kinetic resolution. The absolute configuration was assigned via comparison of the specific optical rotation of the isolated and purified reaction product.

In an Erlenmeyer flask (100 mL), paMsr CFE (650 mg) was weighted and dissolved in KPi buffer (50 mM, pH 7.5). The enzyme was allowed to rehydrate for 45 min at 30°C and 130 rpm. Then, DTT (40 mM, solid, 413.9 mg) and *rac*-**9a** (20 mM, 1.3 mmol dissolved in 650 µL MeOH) were added before the suspension was incubated over night (30 °C, 130 rpm). For work-up, the suspension was divided into two plastic tubes (50 mL) and extracted with EtOAc (3x 15 mL each). The organic phases were combined, and the solvent removed under reduced pressure. The product mixture was purified with flash column chromatography (silica gel 60 M, particle size 40-63 µm, 230-400 mesh) eluting the corresponding sulfide **9b** [R_f= 0.92 (EtOAc)] and undetermined side products [R_f=0.55(EtOAc)] with EtOAc. The product (*R*)-**9a** was then obtained by applying EtOAc/MeOH 4/1 [R_f=0.59 (EtOAc/MeOH 4/1; R_f=0.08 (EtOAc)].

(R)-**9a**: white solid, 135 mg yield, 0.58 mmol, 90%, *ee* 97%; $[\alpha]_D^{20} = -57.4$ (c = 2, acetone) (lit.[10] $[\alpha]_D^{25} = -62.5$ (c = 1, acetone); **^{1}H NMR** (300 MHz, $CDCl_3$) δ 2.87-2.58 (m, 2H), 2.56 (s, 3H), 1.76 (quintet, J=7.5 Hz, 2H), 1.45 (td, J=14.0, 7.6 Hz, 2H), 1.31 (s br, 16 H), 0.88 (t, J=6.6 Hz, 3 H) ppm; **^{13}C NMR** (75 MHz, $CDCl_3$) δ 54.81, 38.56, 31.89, 29.58 (2C), 29.51, 29.33, 29.31, 29.19, 28.80, 22.66, 22.55, 14.09 ppm.

6. Supplementary data

6.1. Kinetic resolution of *rac*-1a

The kinetic resolution of *rac*-**1a** was performed using cell free extract (CFE) of methionine sulfoxide reductase from *Pseudomonas alcaliphila*[5] (paMsr), *Pseudomonas montelii*[6] (pmMsr) and *Escherichia coli*[7] (MsrA) as well as purified paMsr, as described in section 2.1 (Figure S9).

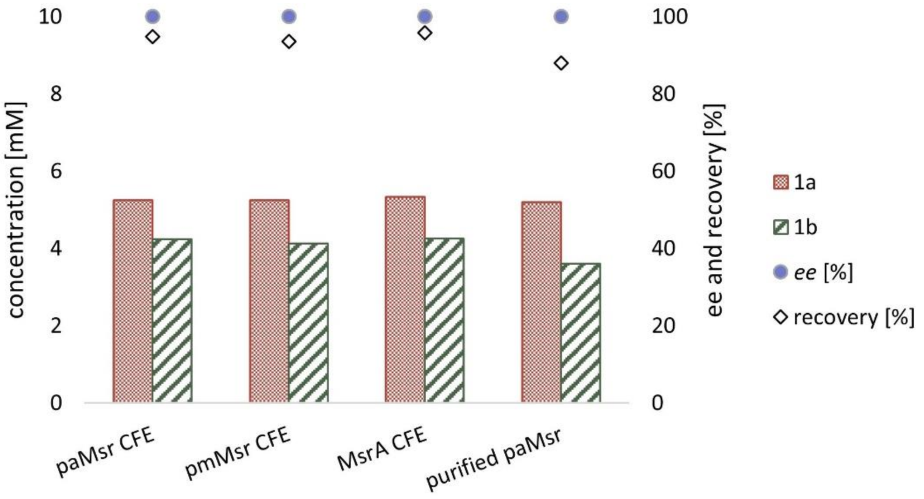

Figure S4. Kinetic resolution of *rac*-**1a** using various biocatalysts. **Reaction conditions:** *rac*-**1a** (10 mM), DTT (10 mM), biocatalyst (5 mg CFE or 0.5 mg purified enzyme), KPi buffer (50 mM, pH 7.5, final volume 0.5 mL), 30 °C, 700 rpm, 1 h.

6.2. Solvent screening of photocatalytic sulfide oxidation with P1

An evaluation of several solvents (H_2O, MeOH, EtOH and ACN) for the photocatalyzed sulfide oxidation using **P1** was performed, applying the procedure as described in section 2.2 (Table S3).

Table S3. Photocatalytic sulfide oxidation by 2-chloro-thioxanthen-9-one (**P1**) in different solvents.

entry	solvent	wavelength [nm]	photocatalyst	conv. [%]
1	H_2O	385	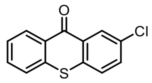 **P1**	30 [a]
2	MeOH	385		71 [a]
3	EtOH	385		69 [a]
4	ACN	385		61 [a]
5	H_2O	385	-	100 [b]
6	H_2O	405[c]	-	<1
7	MeOH	385	-	<1 [a]

[a] reaction time 18 h, [b] reaction time 24 h, [c] illumination at 405 nm, 0.99 µmol photons s^{-1}.
Reaction condition s: **1b** (10 mM), **P1** (0.2-0.4 mg), solvent up to 1 mL reaction volume, 385 nm, 0.75 µmol photons s^{-1}, 25 °C, 500 rpm. 18-24 h.

6.3. Deracemization without photocatalyst at 385 nm

In order to evaluate the stability of the enzyme at 385 nm, purified paMsr and *rac*-**1a** were incubated for 8h under illumination at 385 nm, prior to the addition of the reducing equivalents (DTT) which is required for the reduction step. Successful reduction to the corresponding sulfide **1b** confirmed that the enzyme was still active (Figure S5).

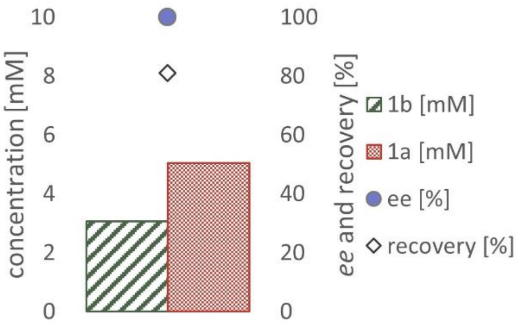

Figure S5. Investigation of the biocatalyst stability under illumination at 385 nm. **Reaction conditions**: *rac*-**1a** (10 mM), DTT after 8 h (10 mM), paMsr (0.5 mg purified), KPi buffer (50 mM, pH 7.5 for a total volume of 0.5 mL), 385 nm, 0.75 µmol photons s^{-1}, 400 rpm, 30 °C.

6.4. Blank and control reactions

Several control reactions were performed (see Table S4) to confirm that the oxidation of the corresponding sulfide intermediate **1b** only occurred in presence of light and photocatalyst (**P6**). The qualitative analysis of the blank and control experiments is based on the quantification of **1a** and **1b** as well as the *ee* of **1a**. Starting from *rac*-**1a**, no oxidation and consequently no deracemization occurred in the absence of either light (entry 4) or photocatalyst (**P6**, pchlide, entry 1) or photocatalyst and methanol (entry 3), while in absence of MeOH and presence of photocatalyst the oxidation was still occurring. MeOH is therefore not responsible for the oxidation. Standard deracemization conditions (section 2.3) were applied for the positive control (entry 5).

Table S4. Blank and control reactions to confirm the light and photocatalyst dependency of the sulfide oxidation and deracemization, respectively.

entry	sample	deracemization	oxidation	reduction	recovery [%]
**1**	w/o pchlide	-	-	+	91
**2**	w/o MeOH	+	+	+	90
**3**	w/o pchlide, w/o MeOH	-	-	+	92
**4**	w/o light	-	-	+	89
**5**	positive	+	+	+	82

+: reaction taking place
-: reaction not taking place
Reaction conditions: **1a** (10 mM), **P6** (14 µM), MeOH (1% v/v), paMsr (30 mg CFE), DTT (20 mM), KPi buffer (50 mM, pH 7.5 for a total volume of 0.5 mL), 455 nm, 0.036 µmol photons s^{-1}, 30 °C, 400 rpm, overnight.

6.5. Light source screening for the deracemization process with P6

Protochlorophyllide (**P6**) shows absorption in the region of 400-480 nm as well as low absorption around 580 nm and 630 nm, respectively. Therefore, several light sources including white light as well as various single wavelengths were evaluated (see Figure S6) in the deracemization process. Similar

results were obtained independent from the applied light sources. For photon and luminous flux see Table S5.

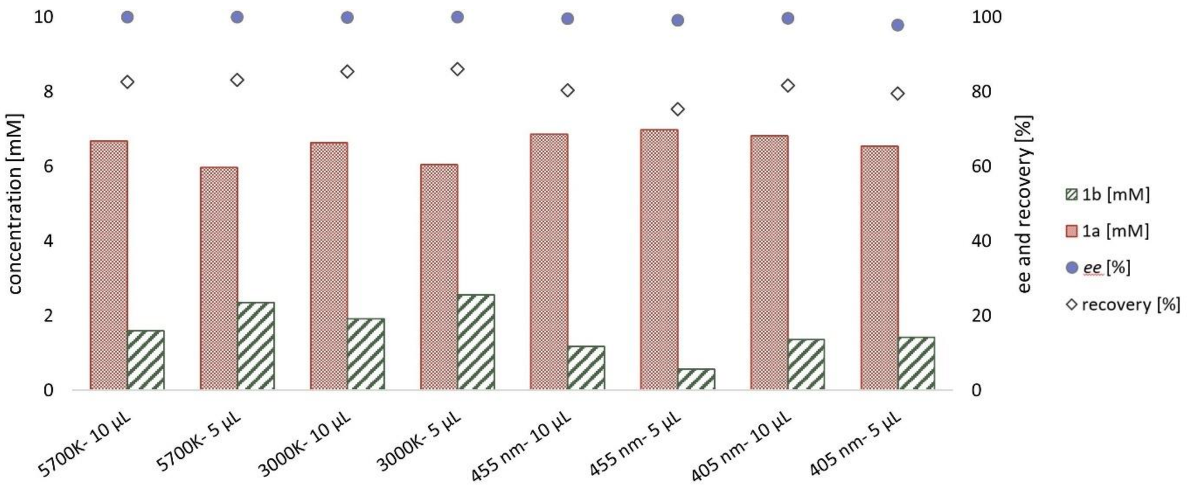

Figure S6. Screening of light sources including white light (5700K, 300K) and single wavelengths (405/455 nm) for the deracemization process with **P6**. **Reaction conditions**: **1a** (10 mM), DTT (20 mM), paMsr (30 mg CFE), MeOH (2% v/v), **P6** (51 µM), KPi buffer (50 mM, pH 7.5, for a total volume of 0.5 mL), 30 °C, 400 rpm, 20 h.

Table S5. Photon and luminous flux for the applied light sources.

entry	light source	effective photon flux [µmol s^{-1}]	luminous flux [lm]
1	5700 K	-	540
2	3000 K	-	474
3	405 nm	0.033	-
4	455 nm	0.036	-

Corresponds to 1% of maximum possible light intensity.

6.6. Protochlorophyllide concentration screening

Examination of the protochlorophyllide (**P6**) concentration in the deracemization process showed a slight improvement at higher concentrations (7 µM *vs* 43 µM, Figure S7).

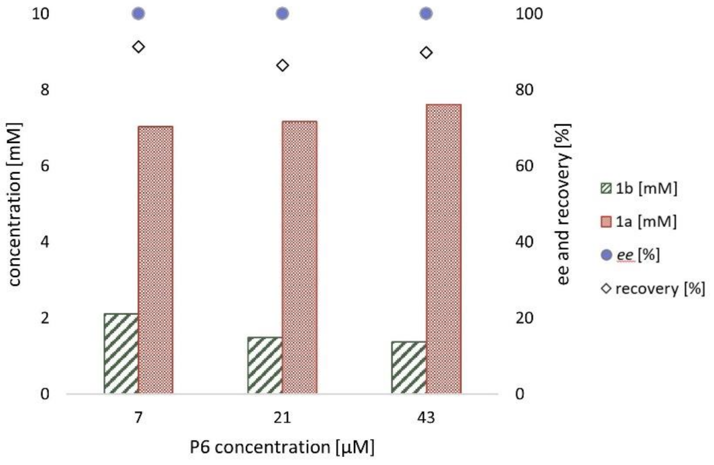

Figure S7. Dependence of the deracemization on the photocatalyst (**P6**) concentration. **Reaction conditions**: *rac*-**1a** (10 mM), DTT (20 mM), paMsr (30 mg CFE), pchlide (7-43 µM), KPi buffer (50 mM, pH 7.5, for a total volume of 0.5 mL), 30 °C, 400 rpm, 455 nm, 0.036 µmol photons s^{-1}, 20 h.

6.7. Redox equivalents in the cell free extract

A kinetic resolution of *rac*-**1a** was performed with and without the addition of external redox equivalents (Figure S8). It is shown that 30 mg CFE of paMsr provides enough redox equivalents for the reduction of the corresponding sulfoxide, since the addition of DTT as external reducing agent did not result in better resulst. However, decreasing the amount of CFE (only 10 mg instead of 30 mg) led to an incomplete kinetic resolution.

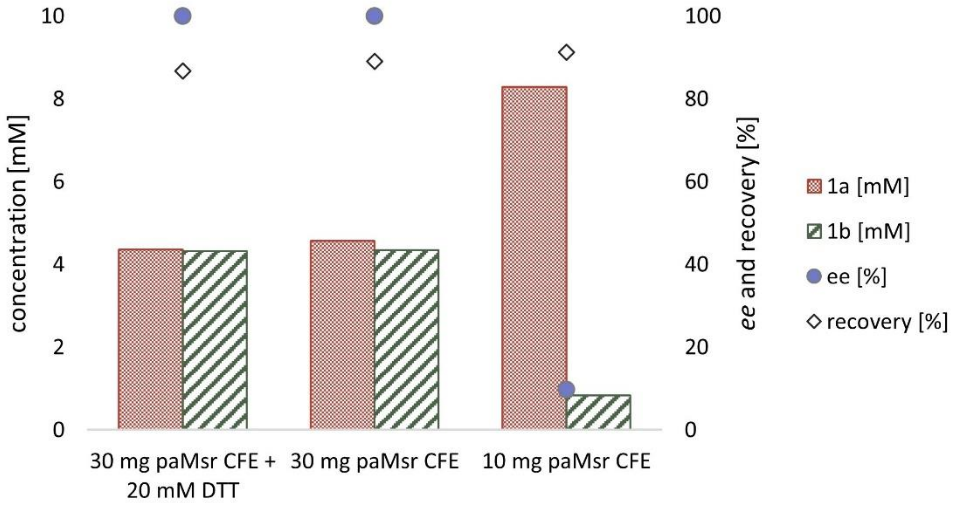

Figure S8. Evaluation of the redox equivalents stored in the CFE in the kinetic resolution of *rac*-**1a**. **Reaction conditions**: *rac*-**1a** (10 mM), DTT (0 or 20 mM), paMsr (10 or 30 mg CFE), KPi buffer (50 mM, pH 7.5, for a total volume of 0.5 mL), 500 rpm, 30 °C, 20 h.

6.8. Time study

The deracemization process of rac-1a was followed over time monitoring the concentration of the sulfoxide **1a**, sulfide intermetiate **1b** and the *ee* of **1a** (Figure S9).

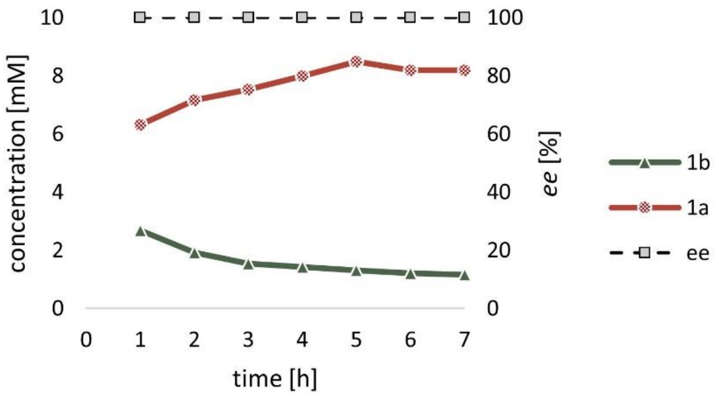

Figure S9. Time course of the deracemization process. **Reaction conditions**: *rac*-**1a** (10 mM), KPi buffer (50 mM, pH 7.5, for a total volume of 0.5 mL), DTT (50 mM), paMsr (5 mg CFE), **P6** (51 µM), 455 nm, 0.036 µmol photons s^{-1}, 400 rpm, 30 °C, 1-7 h, recoveries ~90%.

6.9. Deracemization using purified paMsr

The deracemization of *rac*-**1a** with purified enzyme (paMsr) revealed that triton X-100 is required to obtain results as good as with CFE. The addition of catalase was not beneficial (Figure S10).

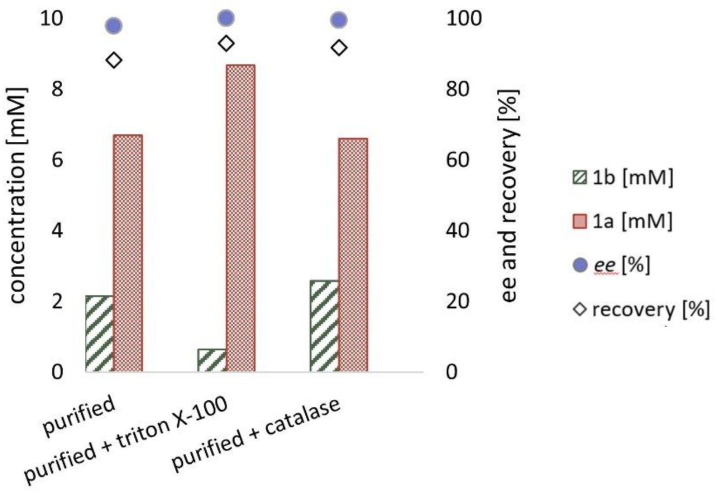

Figure S10. Deracemization of *rac*-**1a** with purified paMsr. **Reaction conditions**: *rac*-**1a** (10 mM), DTT (50 mM), paMsr (0.5 mg purified), **P6** (51 µM), KPi buffer (50 mM, pH 7.5, for a total volume of 0.5 mL), triton X-100 (0.02% v/v) or catalase (4 mg), 455 nm, 0.036 µmol photons s^{-1}, 400 rpm, 30 °C, 24 h.

6.10. pH screening in deracemization

In order to examine the influence of the pH in the deracemization process, various pH values ranging from 5.8 to 7.5 were applied. Lower pH values revealed to be beneficial resulting in less sulfide intermediate **1b** (Table S6).

Table S6. pH value screening.

entry	pH	*ee* [%]	1b [%]
1	5.8	>99	1.7
2	6.0	>99	2.0
3	6.5	>99	4.4
4	7.0	>99	6.5
5	7.5	>99	9.2

Reaction conditions: KPi buffer (50 mM, pH 5.8-7.5, for a total volume of 0.5 mL), *rac*-**1a** (10 mM), paMsr (5 mg CFE), DTT (50 mM), **P6** (51 µM), 455 nm, 0.036 µmol photons s^{-1},400 rpm, 30 °C, 20 h. Recoveries were >90%.

7. Analytical methods

Table S7. GC-FID method and retention times for the analysis of **1a**-**1c** and **9a**.

entry	compound	column	retention time T_R/[min]		program
1	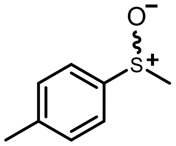 **1a**	Rt®-BDEXse<br>30 m<br>0.32 mmID<br>0.25 µmdf	**1b**<br>(*S*)-**1a**<br>(*R*)-**1a**<br>**1c**	6.7<br>15.0<br>15.1<br>17.6	split ratio 10:1; 2 mL/min gas flow. Temperature program: 100 °C hold 1 min, 7 °C/min to 127 °C hold 3 min, 7 °C/min to 170 °C hold 3 min, 10 °C/min to 180 °C hold 1 min.
2	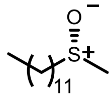 **9a**	Rt®-BDEXse<br>30 m<br>0.32 mmID<br>0.25 µmdf	(*S*)-**1a**<br>(*R*)-**1a**	75.8<br>77.4	split ratio 10:1; 2.5 mL/min gas flow.<br>Temperature program: 80 °C hold 1 min, 20 °C/min to 150 °C hold 81 min, 20 °C/min to 200 °C hold 1 min.

Table S8. HPLC methods for the analysis of **2a**-**8a** and **10a**.

entry	compound	column	retention time T_R/[min]		program	ref.
1	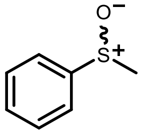 **2a**	OD-H Chiralcel	(*R*)-**2a**<br>(*S*)-**2a**	16.3<br>21.2	heptane/propan-2-ol (93:7)<br>flow rate 1 mL/min<br>oven temperature 25 °C<br>detection at 254 nm	[5]
2	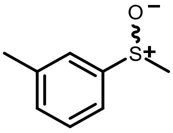 **3a**	IC chiralpak	(*R*)-**3a**<br>(*S*)-**3a**	17.4<br>20.9	heptane/propan-2-ol (75:25)<br>flow rate 1 mL/min<br>oven temperature 25 °C<br>detection at 254 nm	[8]
3	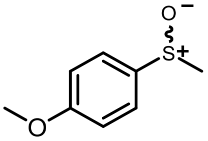 **4a**	OD-H Chiralcel	(*R*)-**4a**<br>(*S*)-**4a**	16.6<br>18.8	heptane/propan-2-ol (90:10)<br>flow rate 1 mL/min<br>oven temperature 25 °C<br>detection at 254 nm	[8]
4	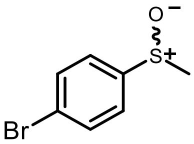 **5a**	IC chiralpak	(*R*)-**5a**<br>(*S*)-**5a**	14.1<br>15.1	heptane/propan-2-ol (75:25)<br>flow rate 1 mL/min<br>oven temperature 25 °C<br>detection at 254 nm	[8]

5	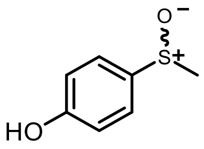<br>**6a**	IC chiralpak	(*R*)-**6a**<br>(*S*)-**6a**	10.0<br>10.7	heptane/propan-2-ol (75:25)<br>flow rate 1 mL/min<br>oven temperature 25 °C<br>detection at 220 nm	[9]
6	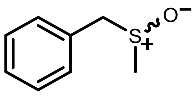<br>**7a**	IA chiralpak	(*S*)-**7a**<br>(*R*)-**7a**	24.3<br>27.0	heptane/propan-2-ol (95:5)<br>flow rate 1 mL/min<br>oven temperature 25 °C<br>detection at 220 nm	[8]
7	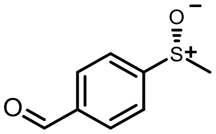<br>**8a**	AD-H chiralpak	(*S*)-8**a**<br>(*R*)-8**a**	21.8<br>23.7	heptane/propan-2-ol (95:5)<br>flow rate 1 mL/min<br>oven temperature 20 °C<br>detection at 230 nm	[5]
8	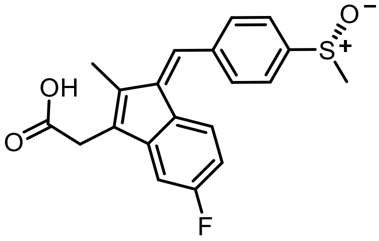<br>**10a**	IC chiralpak	(*S*)-8**a**<br>(*R*)-8**a**	25.6<br>28.7	heptane/propan-2-ol/TFA (75:25:0.1)<br>flow rate 1 mL/min<br>oven temperature 25 °C<br>detection at 220 nm	[11]

8. Calibration curves

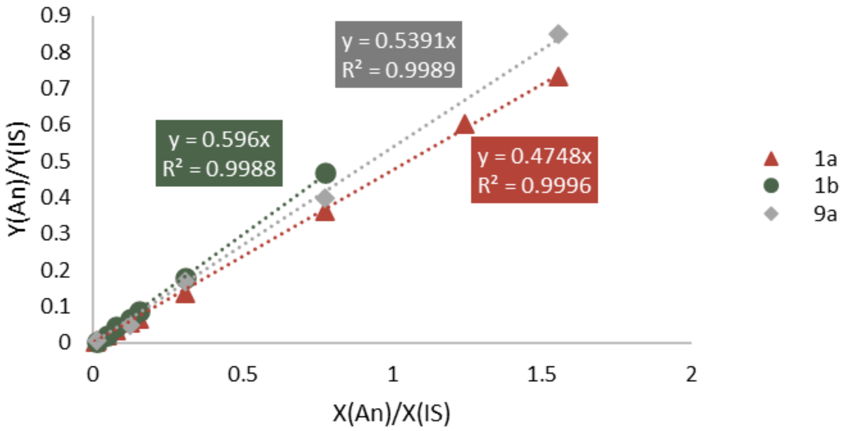

Figure S11. Calibration of **1a** (0.05-10 mM), **1b** (0.1-5 mM) and **9a** (0.1-10 mM) on GC-FID with internal standard (decane, 10 mM).

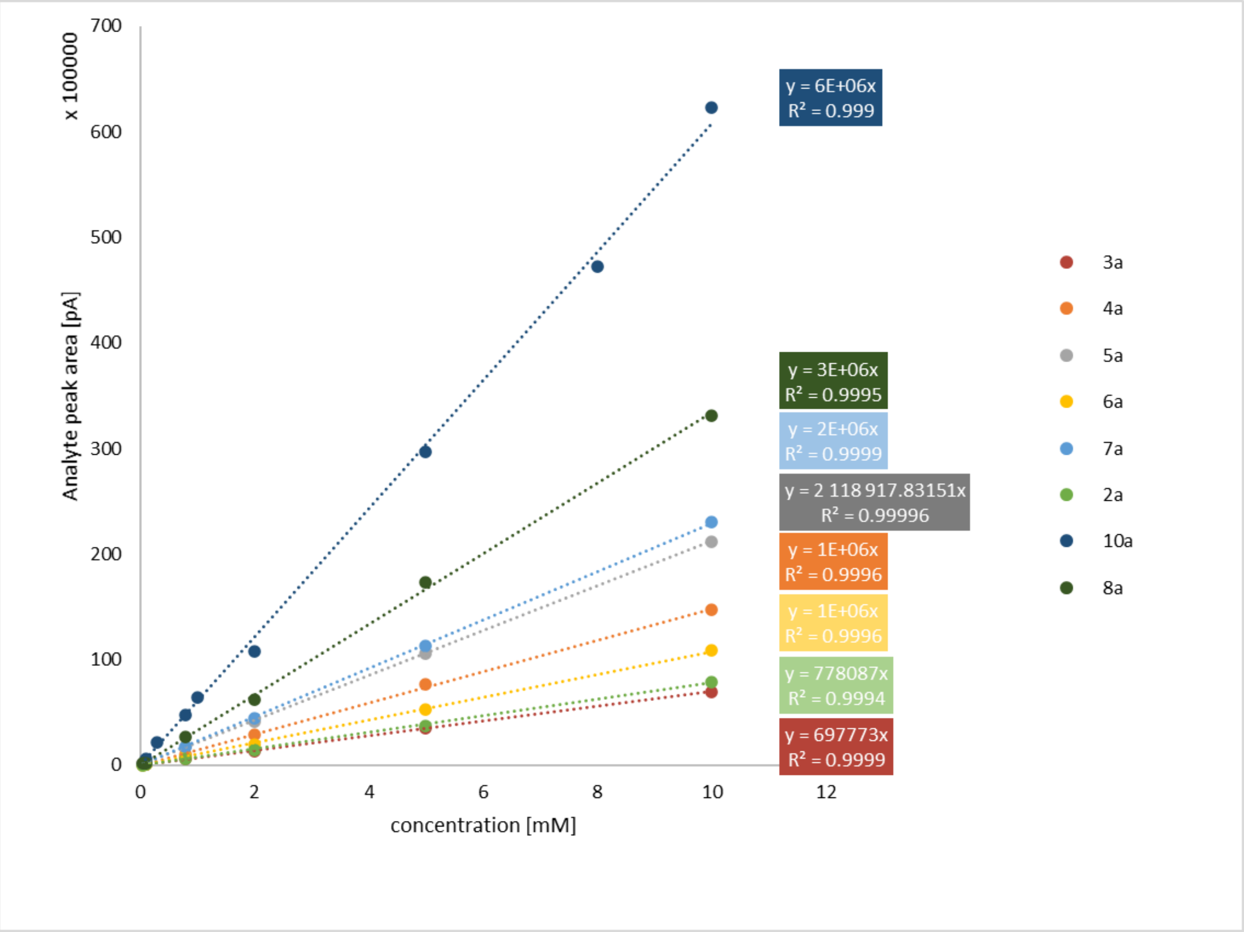

Figure S12. Calibrations of substrates **2a**-**8a** and **10a** using HPLC.

9. GC-FID chromatograms

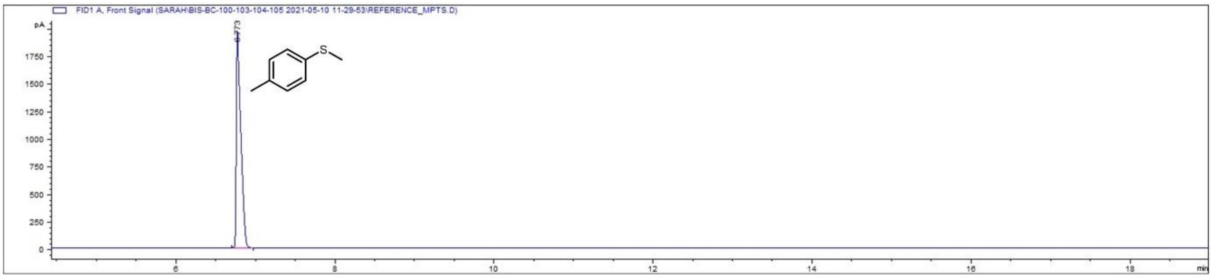

Figure S13. Reference material of **1b** (methyl-*p*-tolyl sulfide).

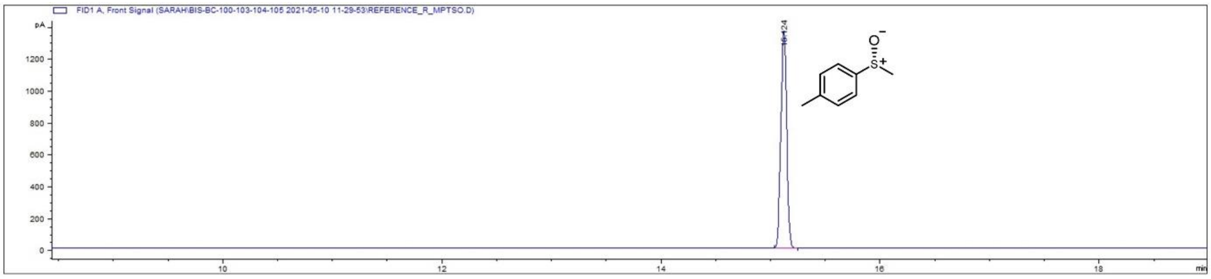

Figure S14. Reference material of (*R*)-**1a.**

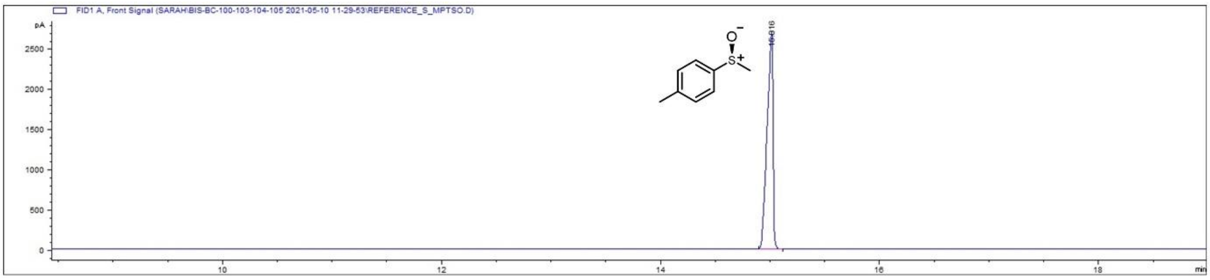

Figure S15. Reference material of (*S*)-**1a.**

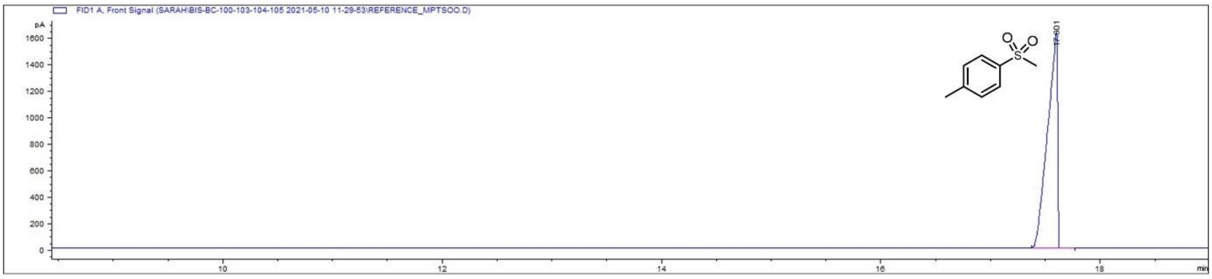

Figure S16. Reference material of **1c** (methyl-*p*-tolyl sulfone).

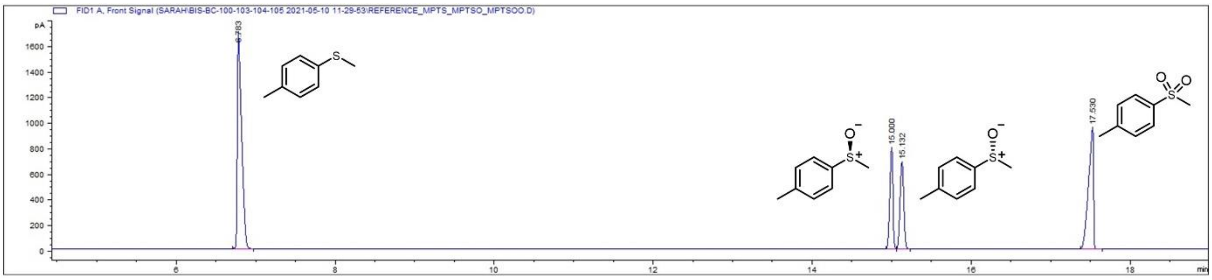

Figure S17. Mixture of **1a**-**1c**.

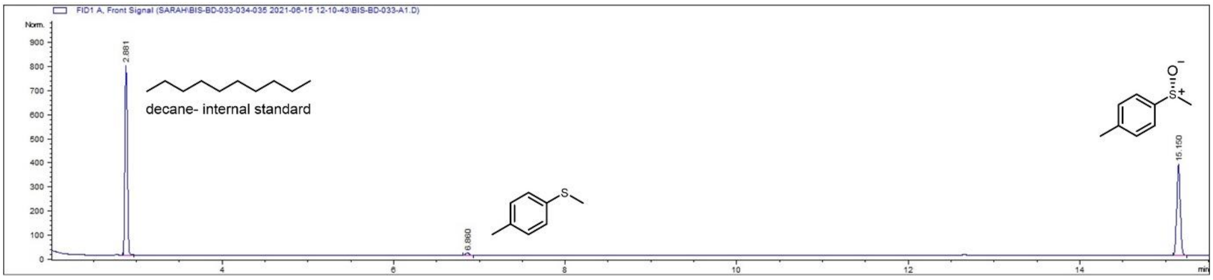

Figure S18. GC chromatogram of an exemplary deracemization of *rac*-**1a** with paMsr (CFE).

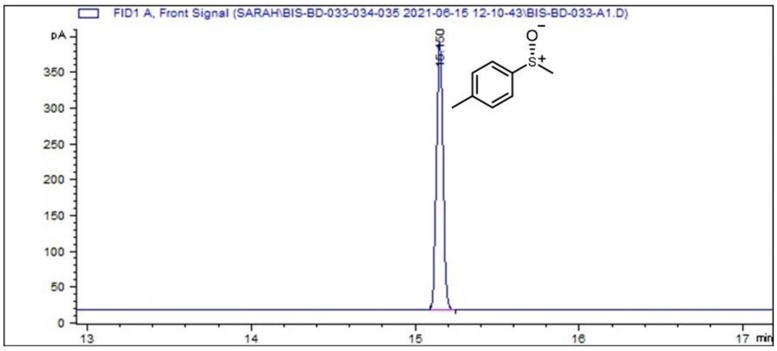

Figure S19. Chiral GC chromatogram of an exemplary deracemization of *rac*-**1a** with paMsr (CFE).

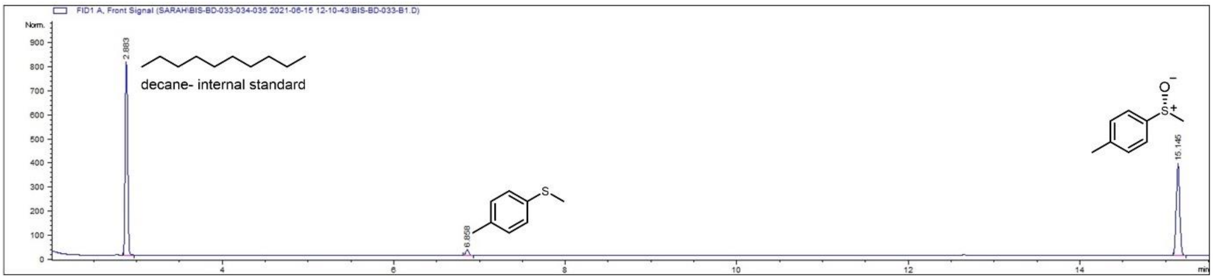

Figure S20. GC chromatogram of an exemplary deracemization of *rac*-**1a** with pmMsr (CFE).

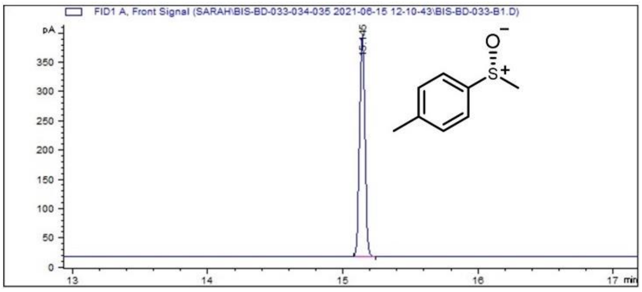

Figure S21. Chiral GC chromatogram of an exemplary deracemization of *rac*-**1a** with pmMsr (CFE).

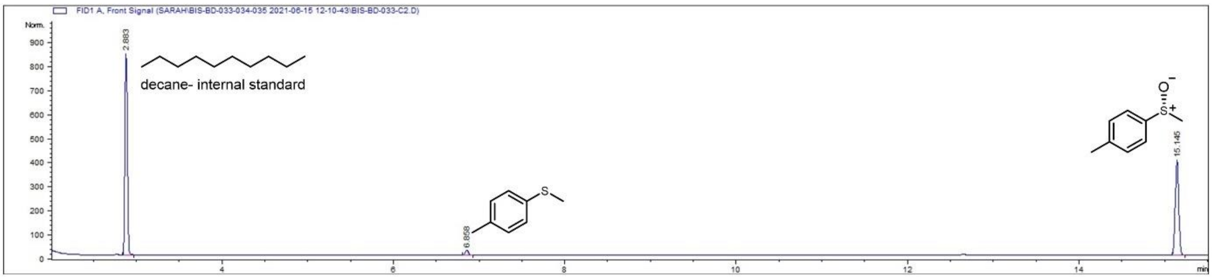

Figure S22. GC chromatogram of an exemplary deracemization of *rac*-**1a** with MsrA (CFE).

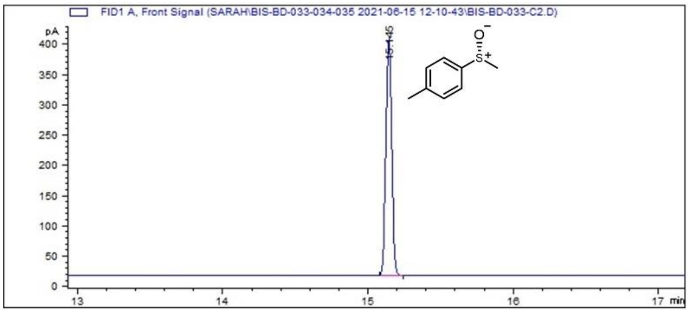

Figure S23. Chiral GC chromatogram of an exemplary deracemization of *rac*-**1a** with MsrA (CFE).

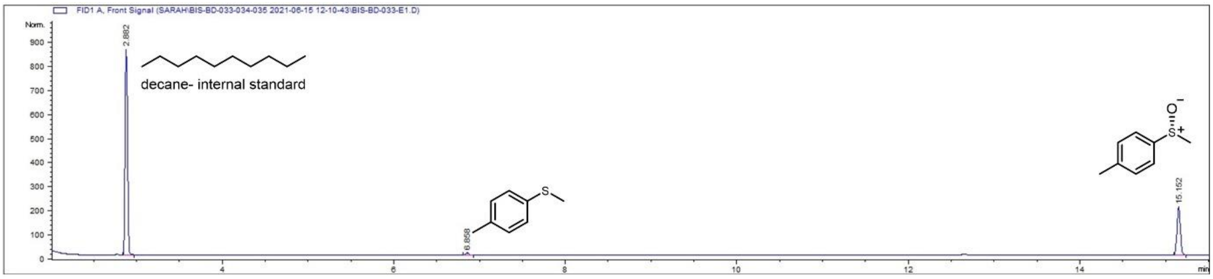

Figure S24. GC chromatogram of an exemplary stereoinversion of (*S*)-**1a** with paMsr (CFE).

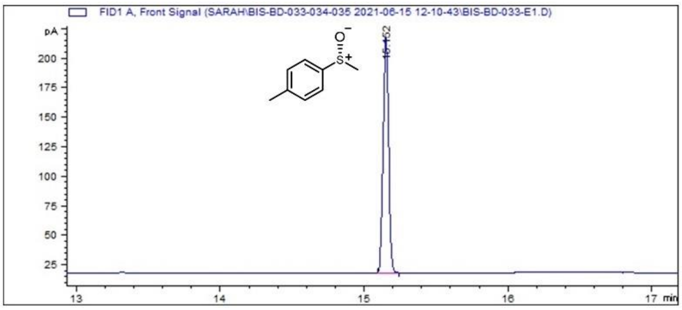

Figure S25. Chiral GC chromatogram of an exemplary stereoinversion of (*S*)-**1a** with paMsr (CFE).

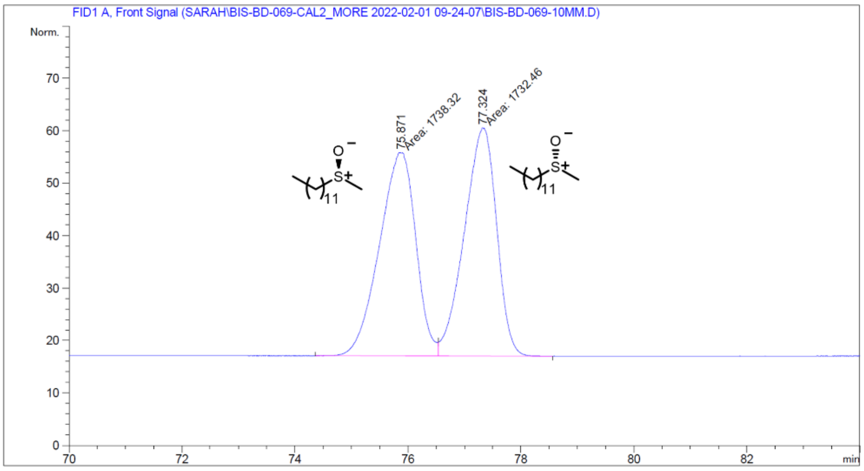

Figure S26. Reference material of *rac*-**9a** (dodecyl methyl sulfoxide).

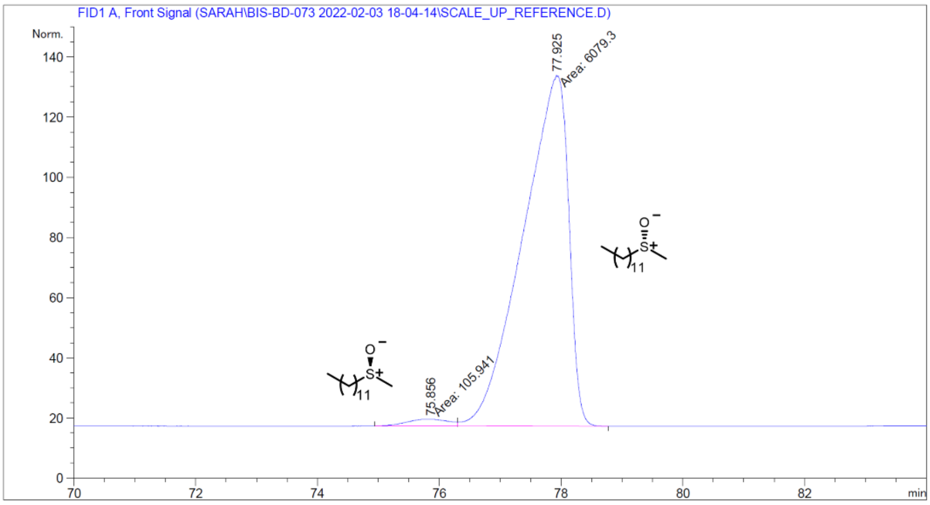

Figure S27. Chiral GC chromatogram of synthesized reference material of (*R*)-**9a** (dodecyl methyl sulfoxide, *ee* 97%).

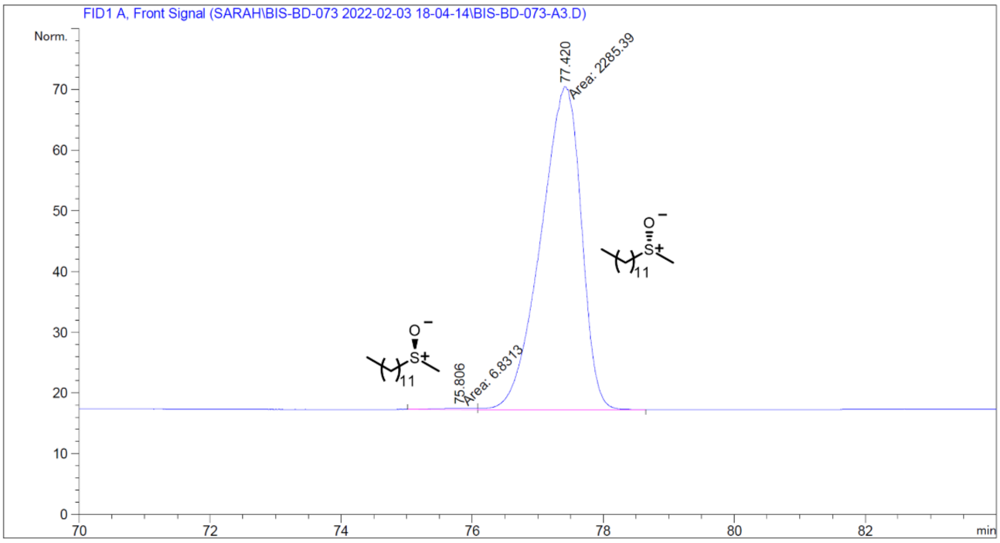

Figure S28. Chiral GC chromatogram of deracemized **9a**.

10. HPLC chromatograms

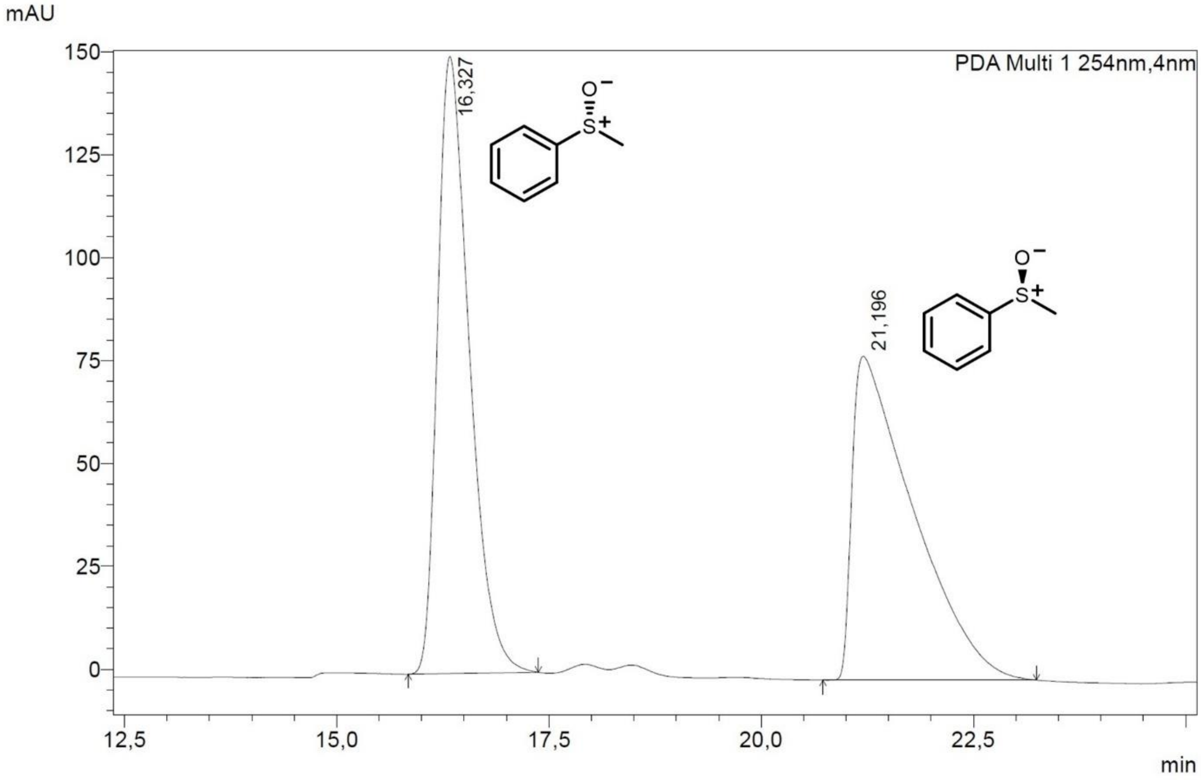

Figure S29. Chiral HPLC chromatogram of *rac*-**2a.**

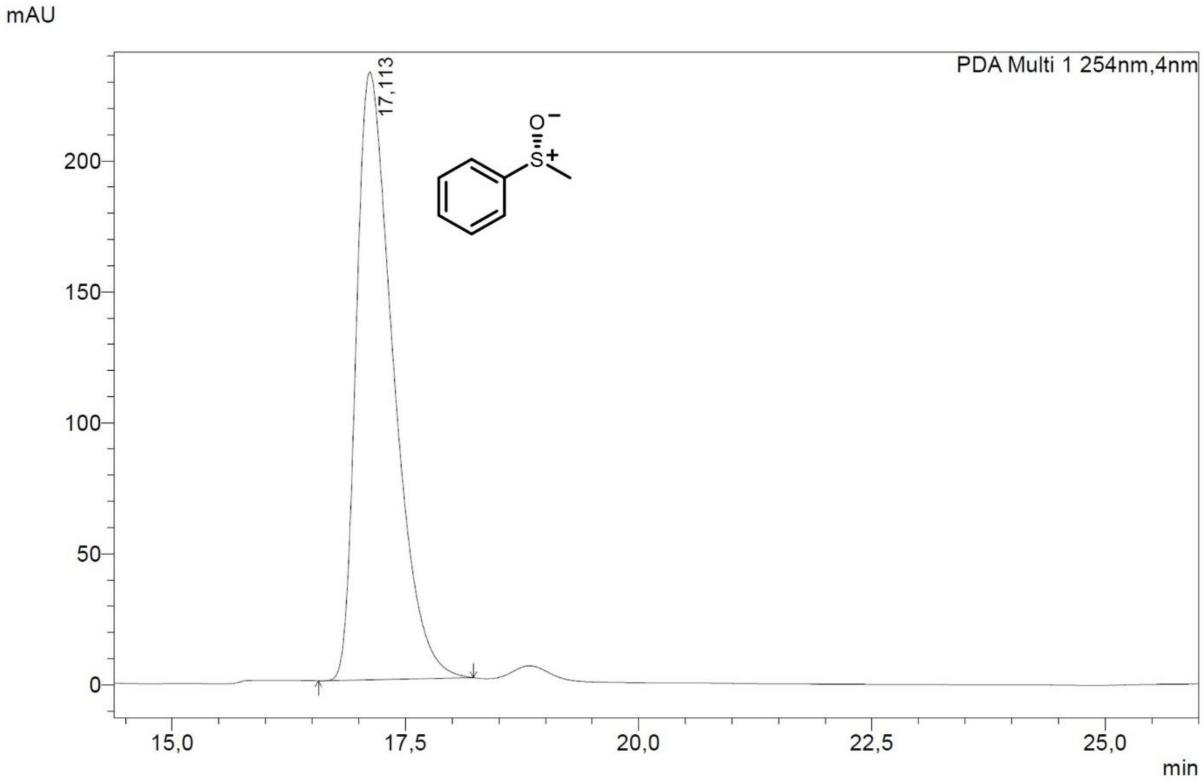

Figure S30. Chiral HPLC chromatogram of deracemized **2a**.

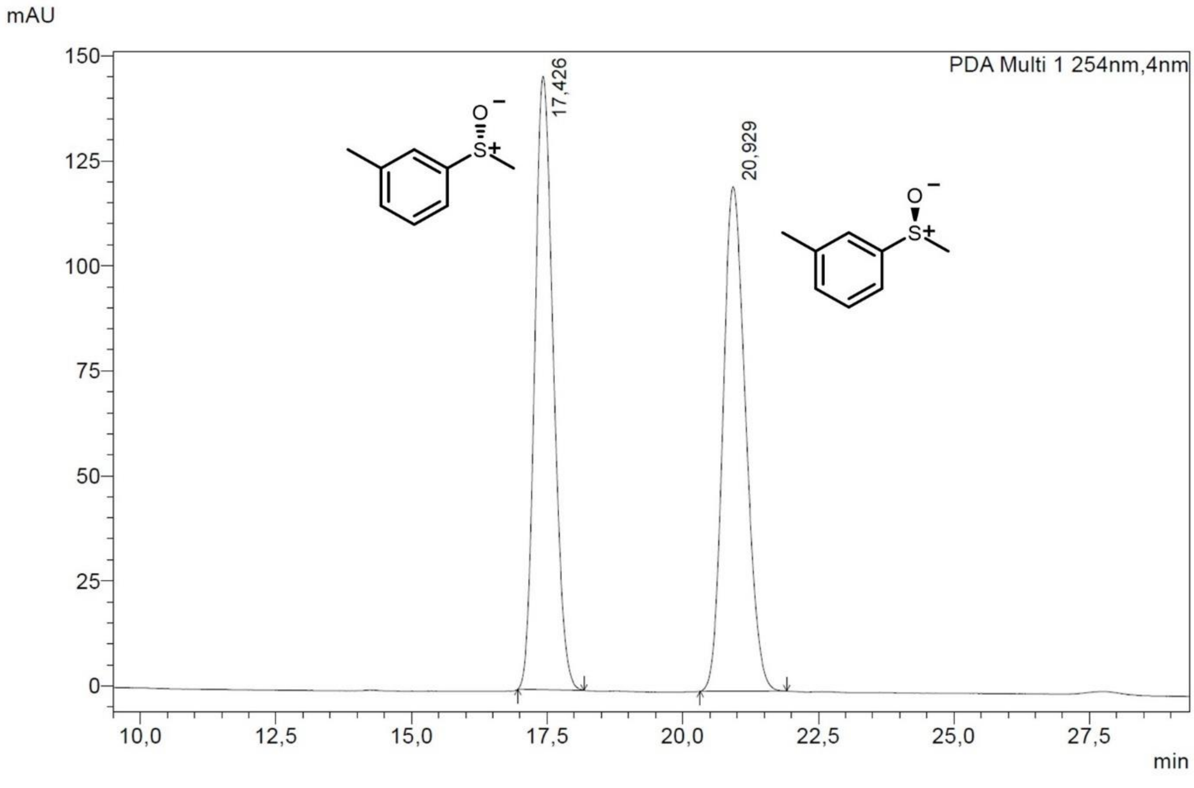

Figure S31. Chiral HPLC chromatogram of *rac*-**3a**.

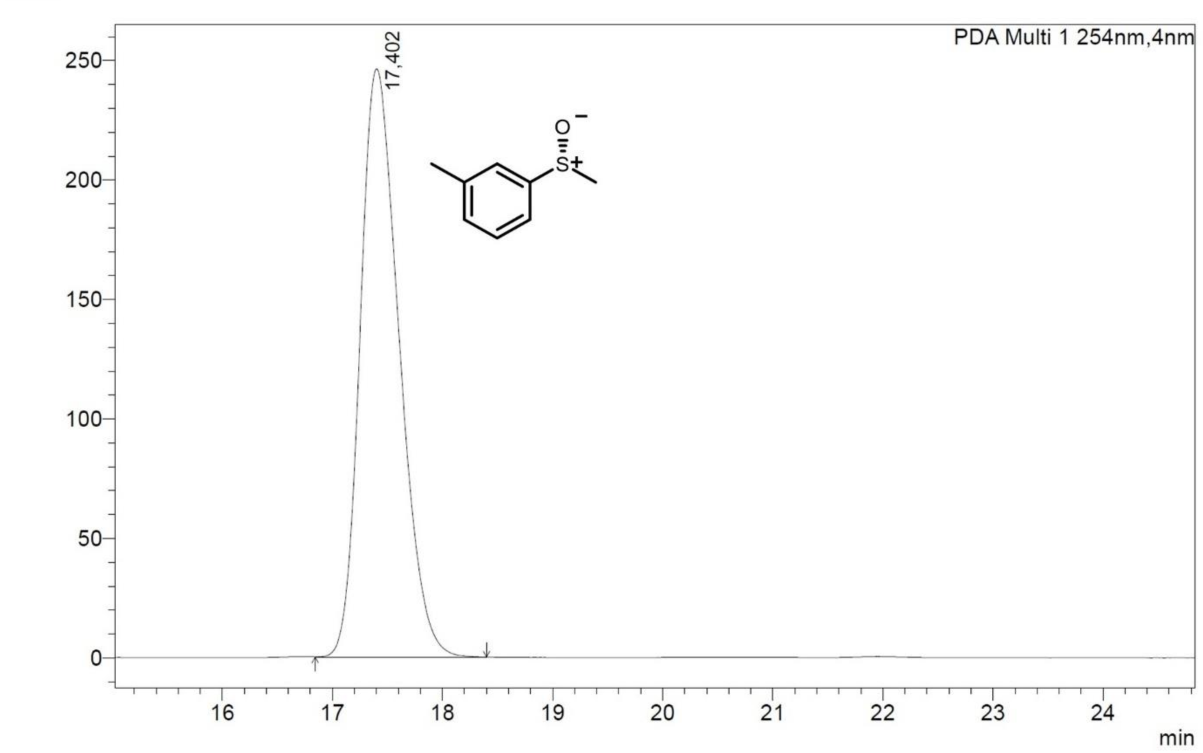

Figure S32. Chiral HPLC chromatogram of deracemized compound **3a**.

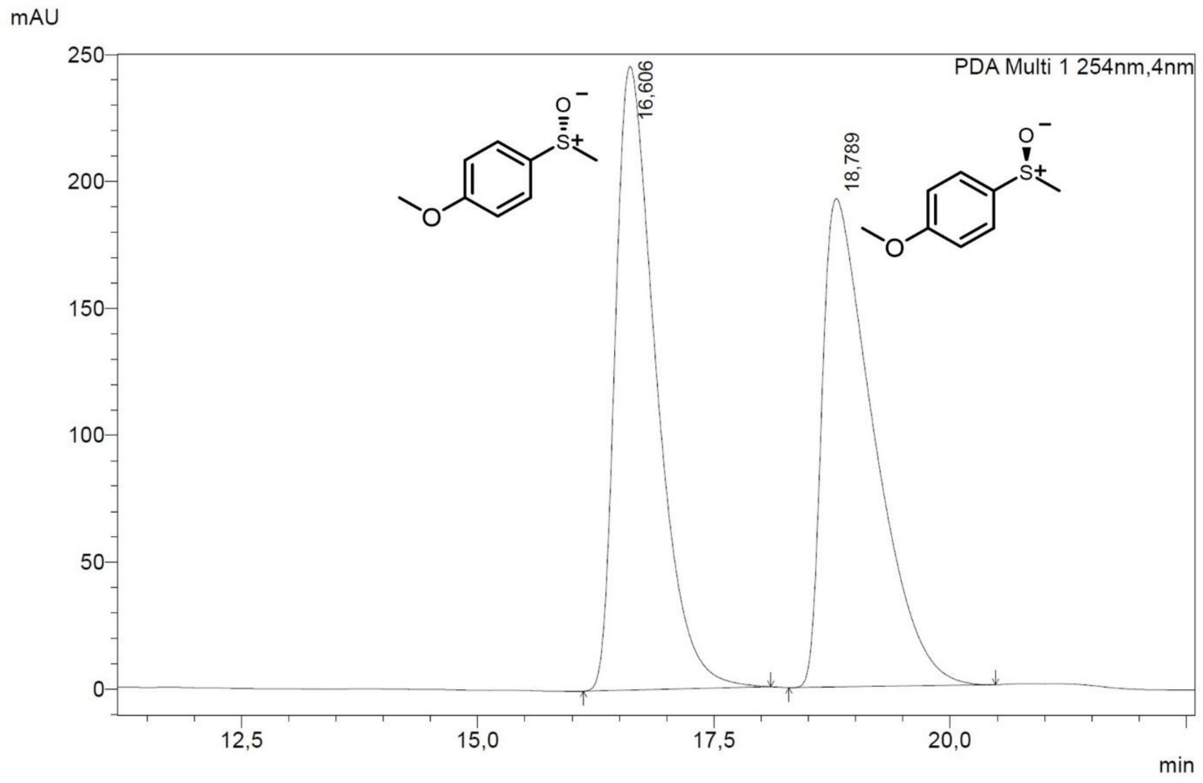

Figure S33. Chiral HPLC chromatogram of *rac*-**4a**.

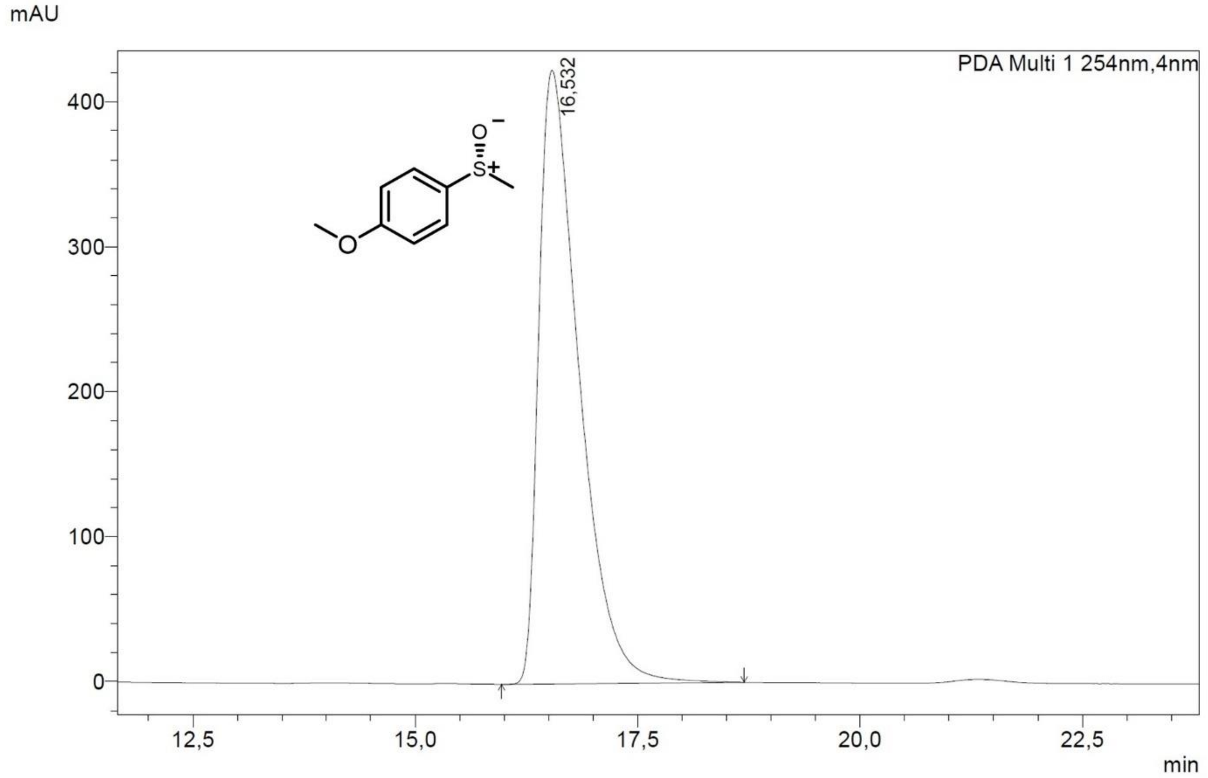

Figure S34. Chiral HPLC chromatogram of deracemized compound **4a**.

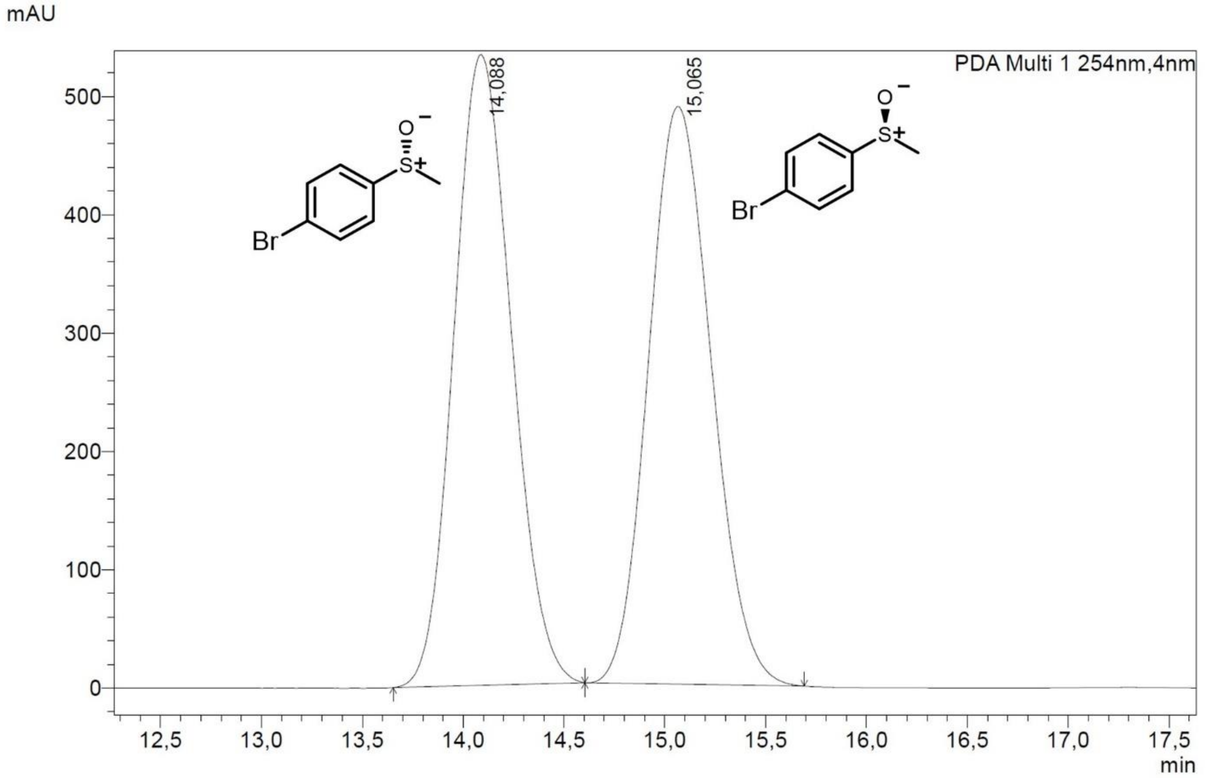

Figure S35. Chiral HPLC chromatogram of *rac*-**5a**.

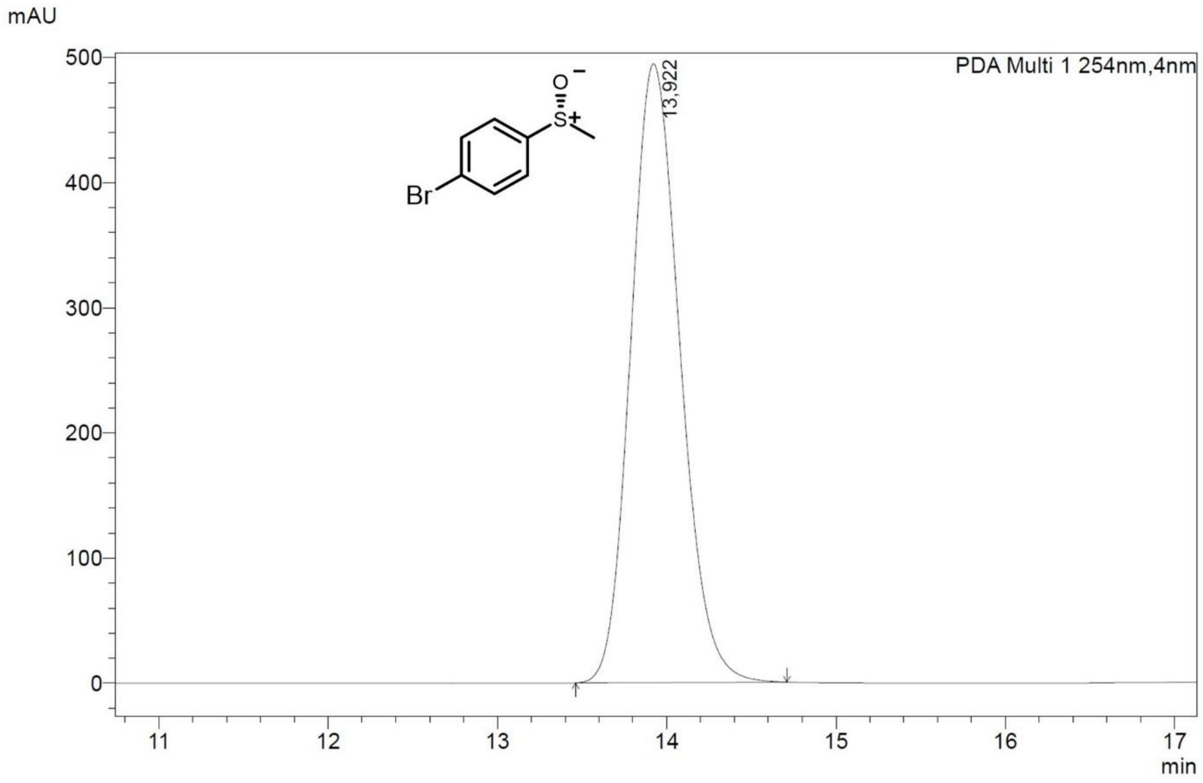

Figure S36. Chiral HPLC chromatogram of deracemized compound **5a**.

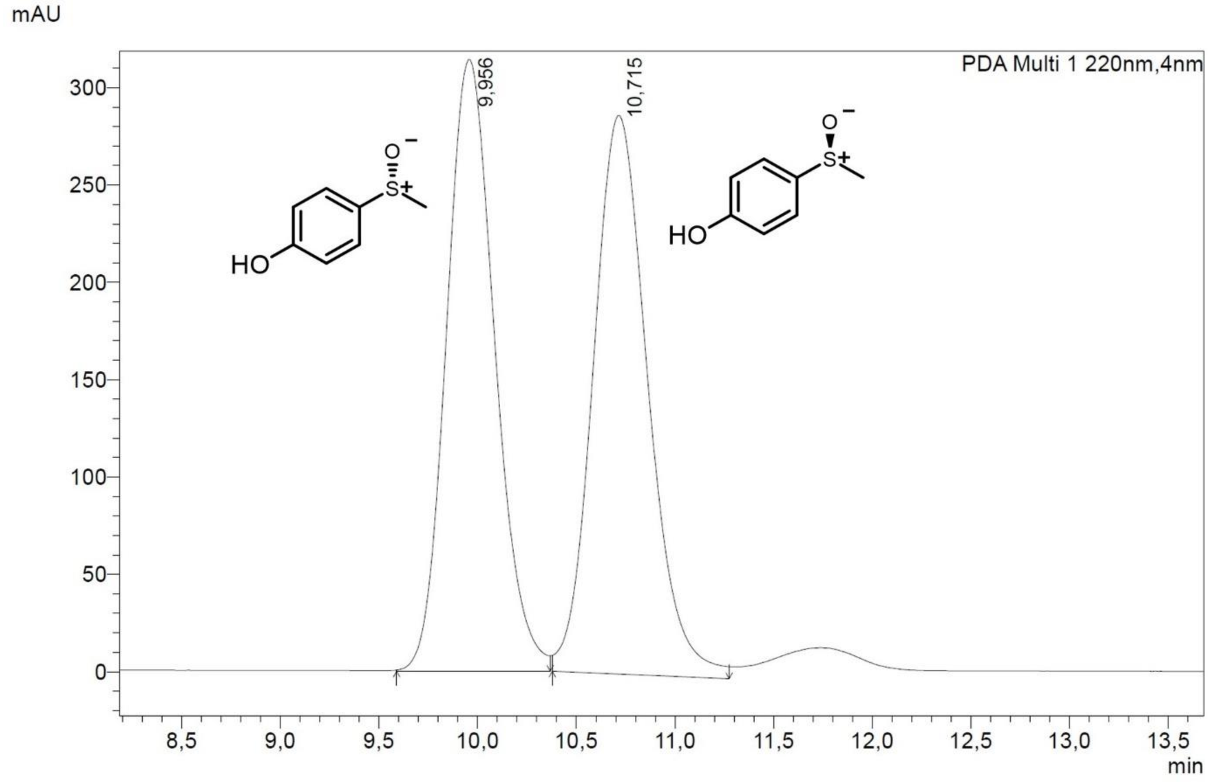

Figure S37. Chiral HPLC chromatogram of *rac*-**6a**.

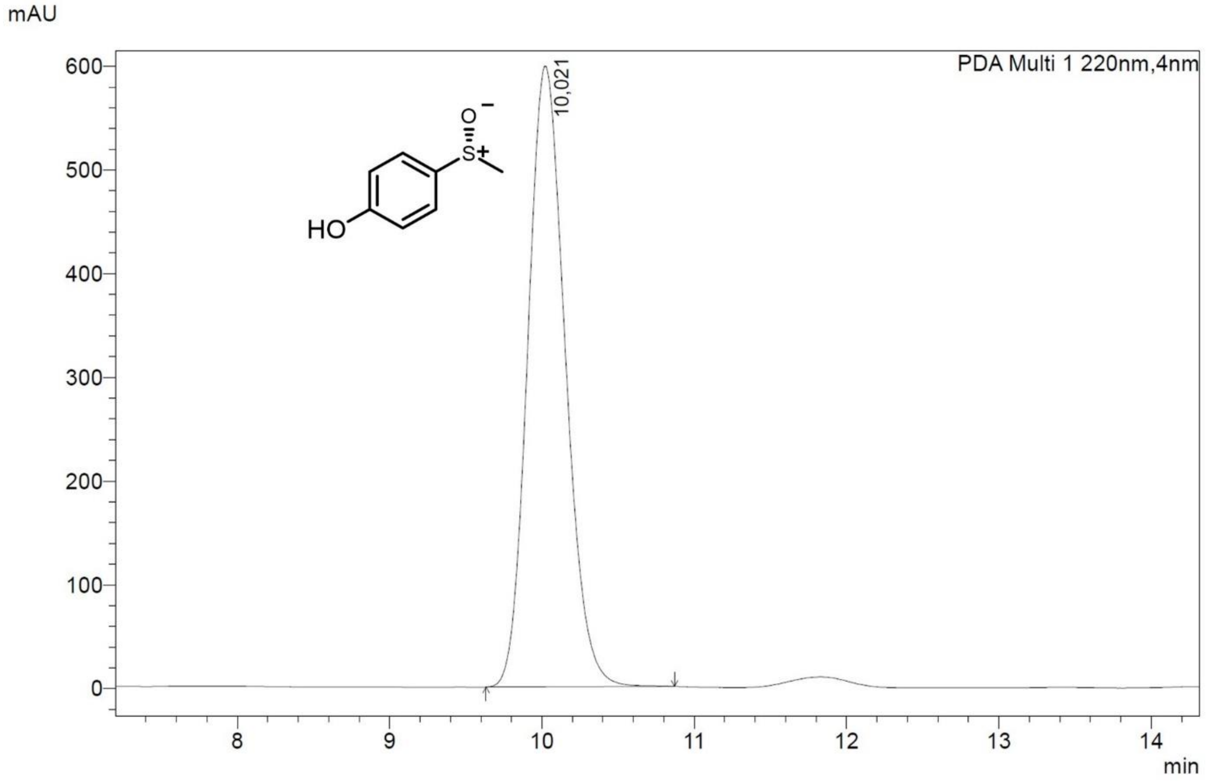

Figure S38. Chiral HPLC chromatogram of deracemized compound **6a**.

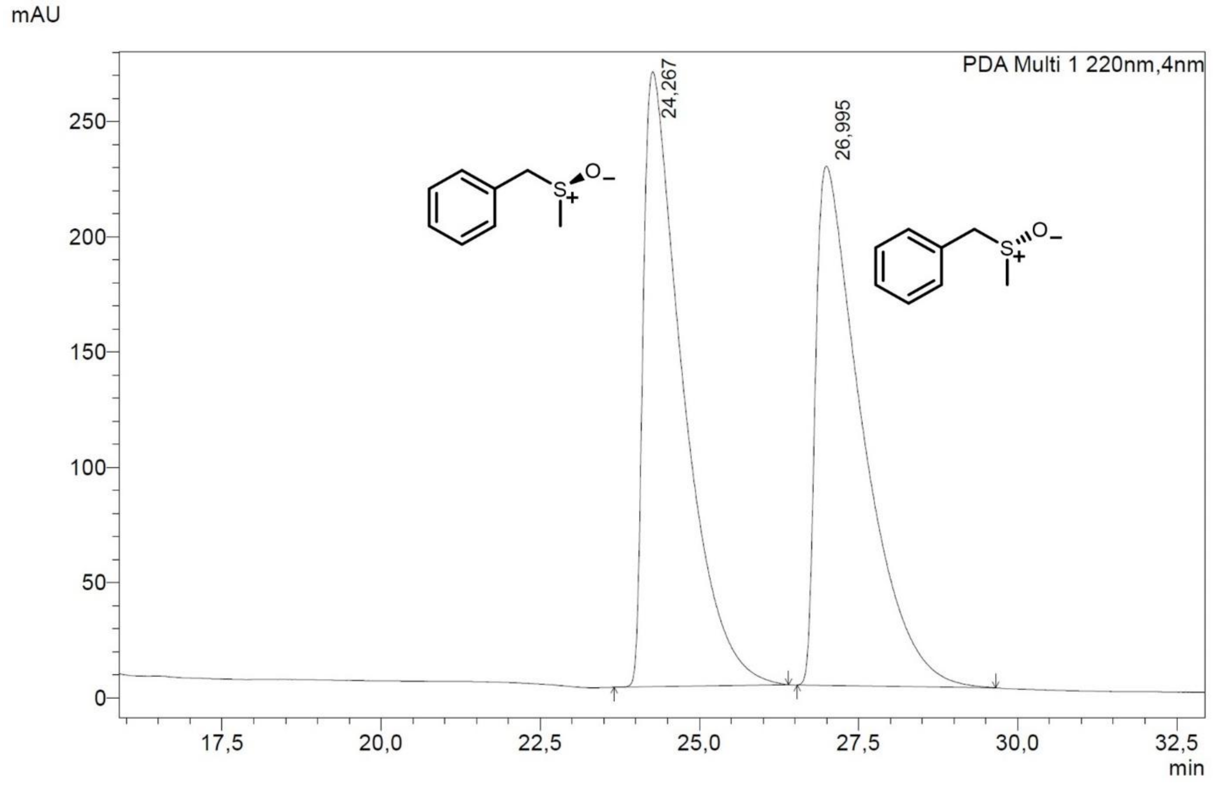

Figure S39. Chiral HPLC chromatogram of *rac*-**7a**.

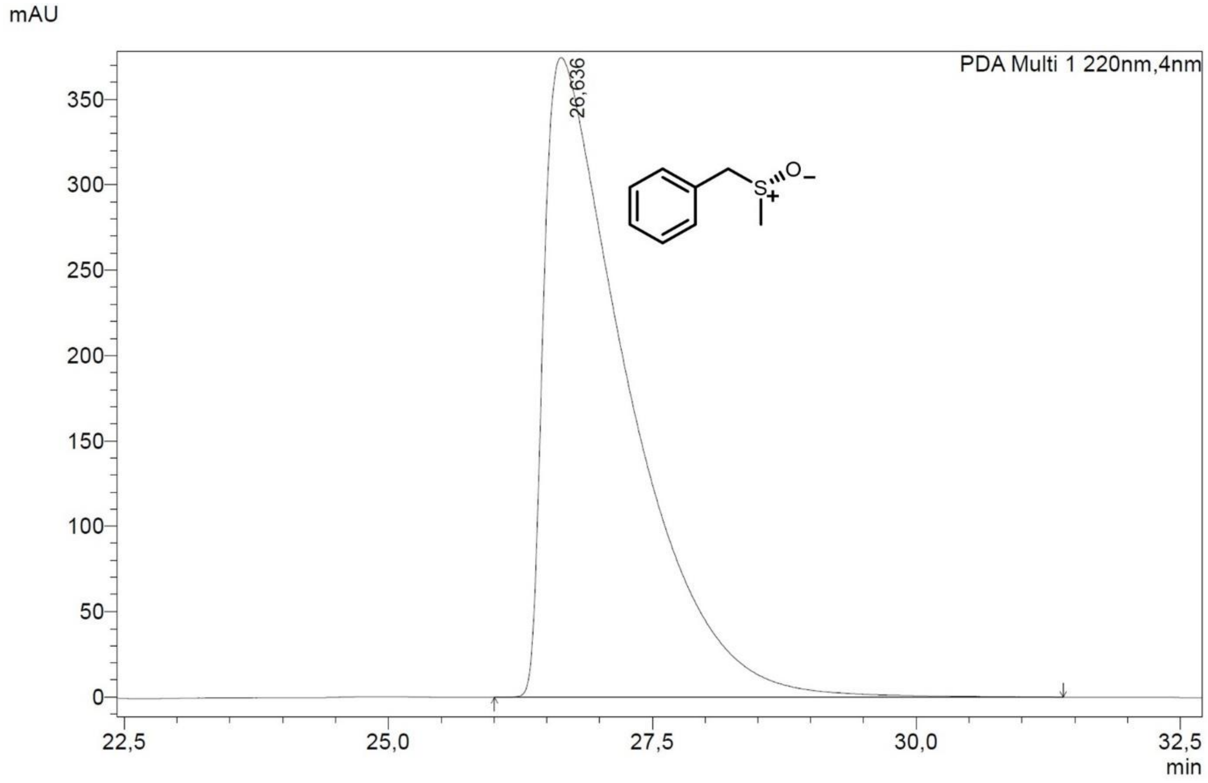

Figure S40. Chiral HPLC chromatogram of deracemized compound **7a**.

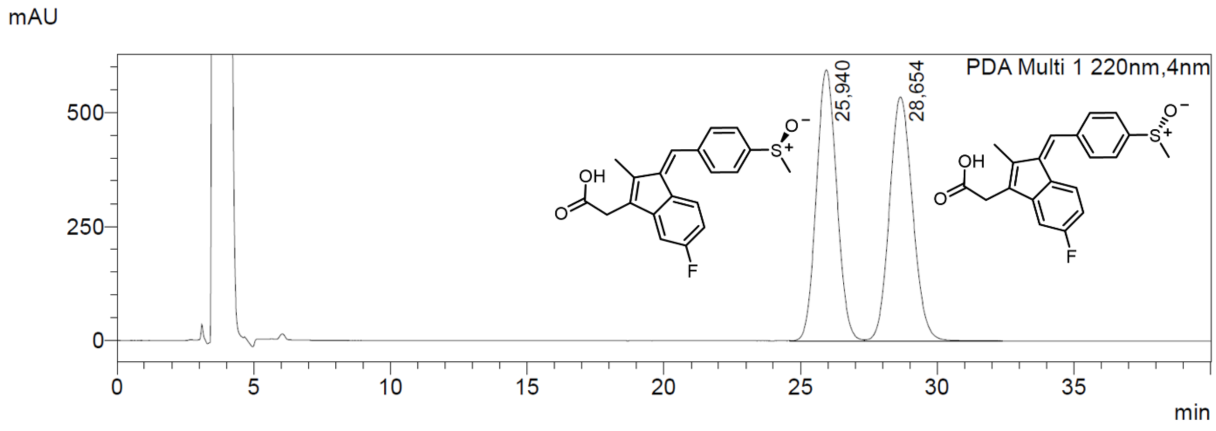

Figure S41. Chiral HPLC chromatogram of *rac*-**10a**.

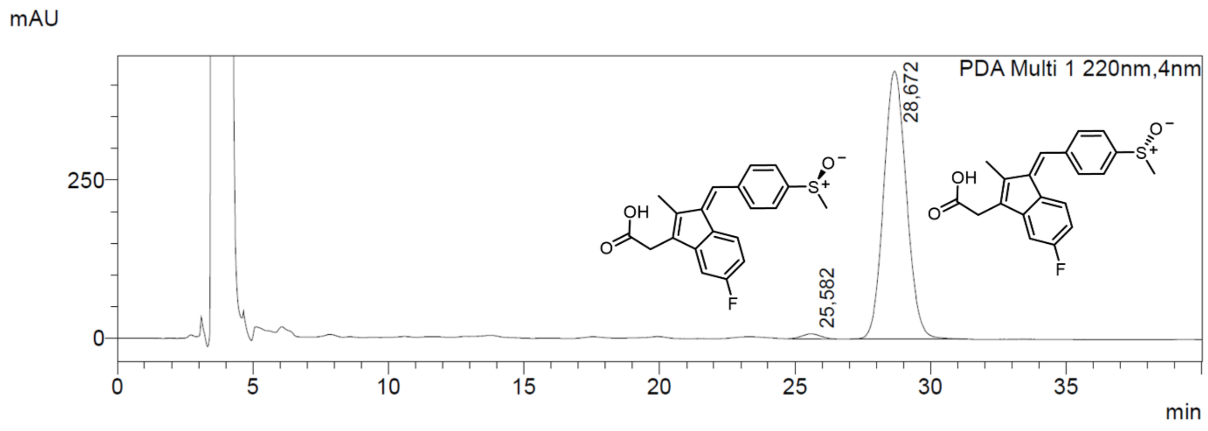

Figure S42. Chiral HPLC chromatogram of deracemized compound **10a**.

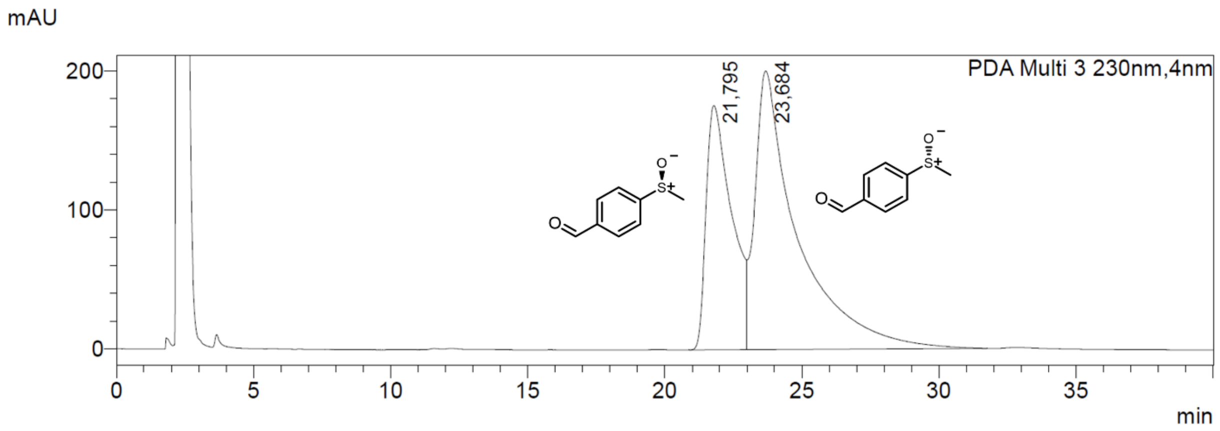

Figure S43. Chiral HPLC chromatogram of *rac*- **8a**.

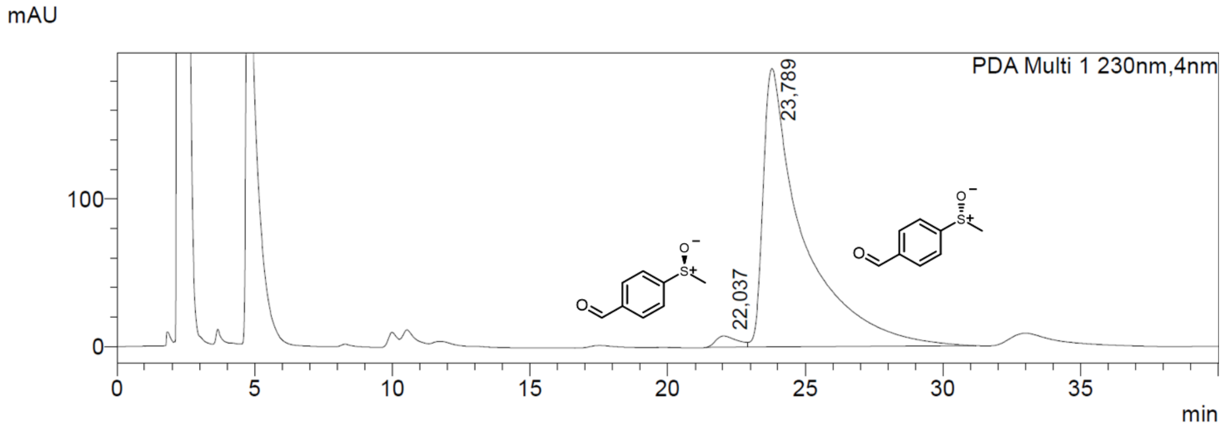

Figure S44. Chiral HPLC chromatogram of deracemized compound **8a**.

11. NMR of (*R*)-**9a**

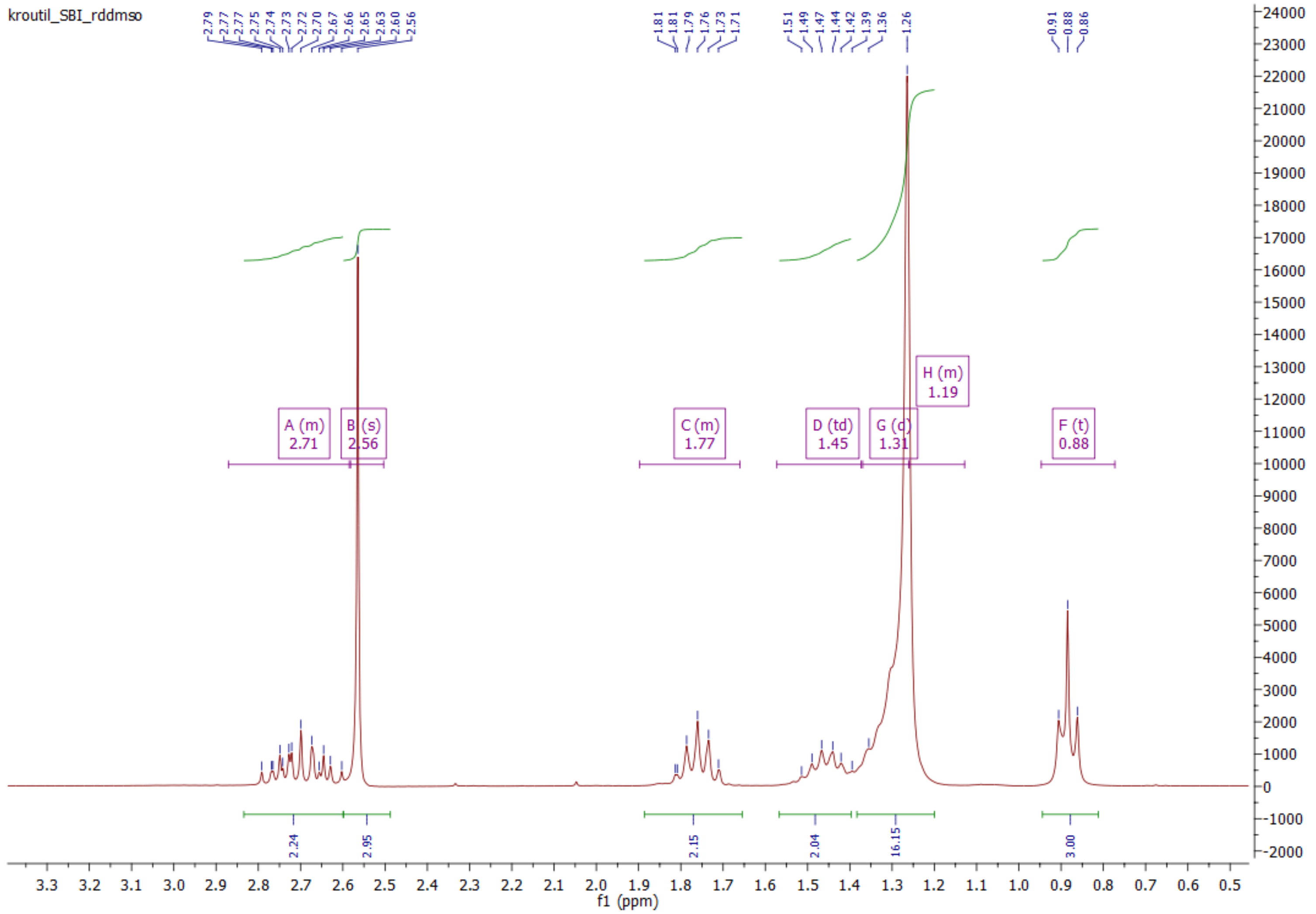

Figure S45. ^{1}H NMR of synthesized (*R*)-**9a** reference compound.

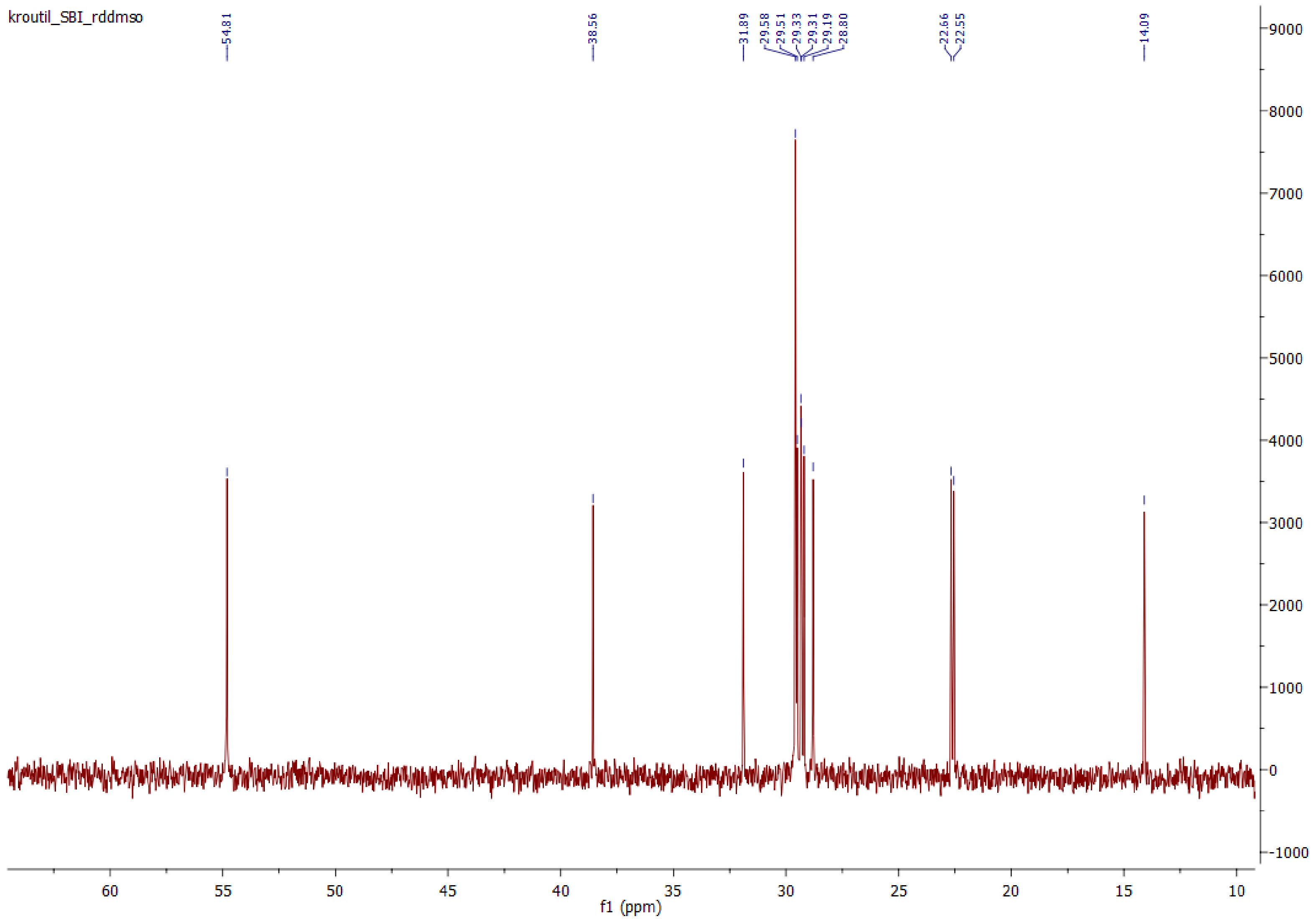

Figure S46. ^{13}C NMR of synthesized (*R*)-**9a** reference compound.

12. Abbreviations

ACN	acetonitrile
API	active pharmaceutical ingredient
CFE	cell free extract
CV	column volume
DMSOR	dimethyl sulfoxide reductase
DTT	dithiothreitol
ee	enantiomeric excess
EtOAc	ethyl acetate
EtOH	ethanol
FID	flame ionisation detector
GC	gas chromatography
HPLC	high performance liquid chromatography
kDa	kilo dalton
MeOH	methanol
Msr	methionine sulfoxide reductase
MsrA	methionine sulfoxide reductase originating from *E. coli*
OD_{600}	optical density at 600 nm
paMsr	methionine sulfoxide reductase originating from *pseudomonas alcaliphila*
PC	photocatalyst
pchlide	protochlorophyllide
pmMsr	methionine sulfoxide reductase originating from *pseudomonas montelii*
rac	racemic
rpm	rounds per minute
RT	room temperature

13. References

[1] C. Winkler, S. Simić, V. Jurkaš, S. Bierbaumer, L. Schmermund, S. Poschenrieder, S. A. Berger, E. Kulterer, R. Kourist, W. Kroutil, *ChemPhotoChem* **2021**, 957.

[2] Y. Xia, K. Li, J. Li, T. Wang, L. Gu, L. Xun, *Nucleic Acids Res.* **2019**, *47*, e15.

[3] a) D. J. Heyes, A. V. Ruban, C. N. Hunter, *Biochemistry* **2003**, *42*, 523; b) L. Schmermund, S. Bierbaumer, V. K. Schein, C. K. Winkler, S. Kara, W. Kroutil, *ChemCatChem* **2020**, *12*, 4044.

[4] H. Klement, M. Helfrich, U. Oster, S. Schoch, W. Rüdiger, *Eur. J. Biochem.* **1999**, *265*, 862.

[5] J. Yang, Y. Wen, L. Peng, Y. Chen, X. Cheng, Y. Chen, *Org. Biomol. Chem.* **2019**, *17*, 3381.

[6] L. Peng, Y. Wen, Y. Chen, Z. Yuan, Y. Zhou, X. Cheng, Y. Chen, J. Yang, *ChemCatChem* **2018**, *10*, 3284.

[7] M. A. Rahman, H. Nelson, H. Weissbach, N. Brot, *J. Biol. Chem.* **1992**, *267*, 15549.

[8] V. Nosek, J. Míšek, *Chem. Commun.* **2019**, *55*, 10480.

[9] V. Nosek, J. Míšek, *Angew. Chem. Int. Ed.* **2018**, *57*, 9849.

[10] C. Cardellicchio, F. Naso, M. A. M. Capozzi, *Tetrahedron: Asymmetry* **2004**, *15*, 1471.

[11] S. Liao, I. Čorić, Q. Wang, B. List, *J. Am. Chem. Soc.* **2012**, *134*, 10765.
